# Composites of Polymer Hydrogels and Nanoparticulate Systems for Biomedical and Pharmaceutical Applications

**DOI:** 10.3390/nano5042054

**Published:** 2015-12-03

**Authors:** Fuli Zhao, Dan Yao, Ruiwei Guo, Liandong Deng, Anjie Dong, Jianhua Zhang

**Affiliations:** Department of Polymer Science and Engineering, School of Chemical Engineering and Technology, Tianjin University, Tianjin 300072, China; E-Mails: zhaofuli@tju.edu.cn (F.Z.); yaodan@tju.edu.cn (D.Y.); rwguo@tju.edu.cn (R.G.); dengliandong@tju.edu.cn (L.D.); ajdong@tju.edu.cn (A.D.)

**Keywords:** hydrogels, nanoparticles, nanocomposite hydrogels, hybrid hydrogels, nanocomposites, biomedical applications

## Abstract

Due to their unique structures and properties, three-dimensional hydrogels and nanostructured particles have been widely studied and shown a very high potential for medical, therapeutic and diagnostic applications. However, hydrogels and nanoparticulate systems have respective disadvantages that limit their widespread applications. Recently, the incorporation of nanostructured fillers into hydrogels has been developed as an innovative means for the creation of novel materials with diverse functionality in order to meet new challenges. In this review, the fundamentals of hydrogels and nanoparticles (NPs) were briefly discussed, and then we comprehensively summarized recent advances in the design, synthesis, functionalization and application of nanocomposite hydrogels with enhanced mechanical, biological and physicochemical properties. Moreover, the current challenges and future opportunities for the use of these promising materials in the biomedical sector, especially the nanocomposite hydrogels produced from hydrogels and polymeric NPs, are discussed.

## 1. Hydrogels and Their Biomedical and Pharmaceutical Applications

Due to the remarkable characteristics, such as flexibility and versatility in fabrication, variety in composition, high tunability in the physical, chemical, and biological properties, high moldability in shape, especially their excellent biocompatibility and similarity to native extracellular matrix (ECM), hydrogels as promising materials are of greatest significance in the biomedical fields and have been most extensively studied in academic and industrial research [1,2,3,4,5,6,7,8]. Hydrogels are a class of materials that present a three-dimensional (3D) network formed from hydrophilic homopolymers or copolymers crosslinked to form insoluble polymer matrices, which are able to absorb a large amount of water or biological fluids. Moreover, some “intelligent” hydrogels could undergo reversible and significant changes in structure, shape, and/or property after being exposed to external physical/chemical/mechanical stimuli, such as pH value, temperature, ionic strength, enzymatic activity, glucose concentration, light, electric field, magnetic field, pressure and solvent composition or a combination of them [9,10,11,12]. Therefore, these stimuli-sensitive are expected to contribute significantly to the exploration and development of next generation of biomaterials for biological and biomedical applications, such as self-regulated and site-specific drug delivery systems, specialized separation systems or bioreactors.

Since Wichterle and Lim first reported a hydrogel for contact lens application in 1960 based on crosslinked poly-2-hydroxy-ethylmethacrylate (PHEMA) hydrogel by use of ethylene glycol dimethacrylate (EGDMA) [13], significant progress has been achieved and a diverse range of polymers have been used for the synthesis and fabrication of hydrogels for various applications. Besides the PHEMA, a great number of synthetic polymers were explored for preparation of hydrogels by polymerization or cross-linking, such as poly(ethylene glycol) (PEG), poly(vinyl alcohol) (PVA), polyacrylic acid (PAA), poly(2-hydroxypropyl methacrylamide) (PHPMA), polyvinyl pyrrolidone (PVP), polyurethanes (PU), polyacrylamide (PAM), poly(dimethylaminoethyl methacrylate) (PDMAEMA) and poly(*N*-isopropyl acrylamide) (PNIPAM) as well as polypeptides and polyesters. The polymeric hydrogels from synthetic sources are advantageous, because they usually possess controllable chemical structure and architecture, degradation rates, and mechanical strengths. However, most of them lack biological cues for biological applications, especially for proliferation of cells and tissue regeneration [2,3,4,6]. Natural hydrogels derived from natural sources, such as chitosan (CS), hyaluronic acid (HA), gelatin (GEL), collagen (COL), alginate (ALG), elastin (ELA), heparin (HEP) and chondroitin sulfate (CRS), are appealing for biological and biomedical applications because of their cell-interactive properties, cell adhesion and cell signaling, as well as biodegradability and excellent biocompatibility [2,3,4,8]. However, their uses are often restricted due to uncontrollable degradation and structure, low mechanical properties, potential immunogenicity and high batch-to-batch variations [2,3,14]. The well designed and tailored hydrogel materials can be obtained by the combination of natural and synthetic polymers, which have resulted in a shift towards the use of hydrogels based on semisynthetic polymers for biological and biomedical applications, such as P(PEG-co-peptides), ALG-g-(PEG-PPO-PEG), P(PLGA-co-serine), COL-acrylate, P(HPMA-g-peptide) and HA-g-PNIPAM [2,3,6,14].

**Figure 1:**
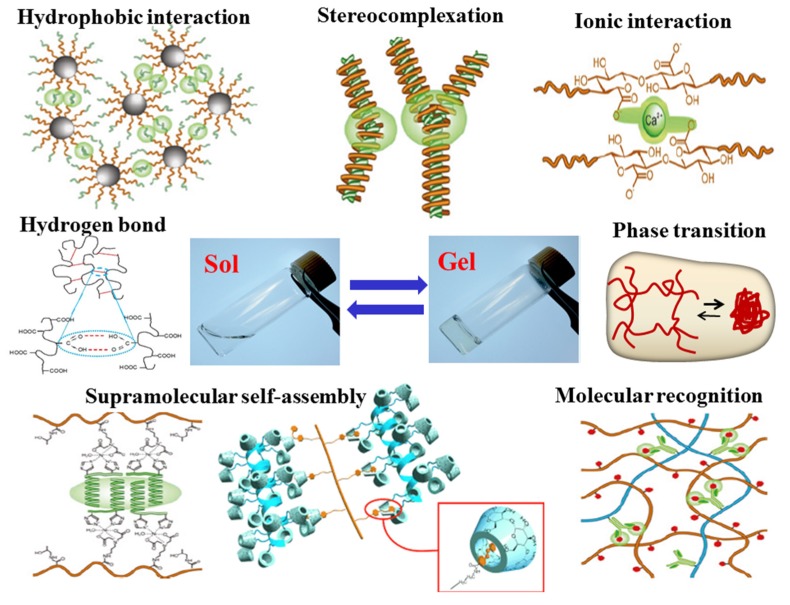
Schematic illustration of methods for formation of physically crosslinked hydrogels.

Briefly, a hydrogel is a crosslinked hydrophilic polymeric network with high amount of water, and thus any techniques which are able to crosslink polymer chains can be applied to prepare a hydrogel. Gelation can take place either by chemical crosslinking (chemical gelation involving formation of permanent covalent bonds) or by physical crosslinking (physical gelation through the emergence of reversible and transient junctions). Chemically crosslinked hydrogels are commonly prepared by radical polymerization of a hydrophilic monomer in the presence of a multifunctional cross-linking agent, or condensation polymerization of monomers with multifunctionalities [3,6,15,16]. Chemical gelation can also occur through covalent bonding between polymeric chains through energy irradiation, chemical reactions or sulphur vulcanization by the addition of different chemicals (crosslinker) [3,6,15,16]. The 3D integrity of hydrogels in the swollen state can also be retained by physical crosslinking through molecular entanglements and/or secondary forces, such as hydrogen bonding, ionic or hydrophobic interactions [3,6,15,17]. Recently, the emergence of bio-orthogonal and supramolecular chemistries provides great opportunities for scientists to create multifunctional physically cross-linked hydrogel matrices [15,17]. All of these interactions are reversible, and thus they can be disrupted by changing physical conditions (pH, temperature, and ionic interactions), as shown in Figure 1. Generally speaking, chemically crosslinked gels often have much higher mechanical properties than their physically crosslinked counterparts. However, the residual organic solvents, chemical crosslinkers, and/or unreacted monomers could leach out from the hydrogels continuously and cause cytotoxicity.

Hydrogels have a variety of properties [3,5,6,17], such as absorption capacity, swelling behavior, stability and degradation, bioadhesion and bioactivity, permeability, mechanical properties optical and surface properties, which make them promising materials for diverse applications. In theory, most of features can be engineered into a hydrogel system [5,17]. Biodegradability of hydrogels is essential for biomedical applications that require controlled resorption *in vivo* or local dissolution to favor cell activities and facilitate tissues reparation. Most of hydrogels are able to undergo local or bulk dissolution by hydrolysis, proteolysis or disentanglement [5,17]. One of the most remarkable characteristics of hydrogels is their highly porous structure, allow for a depot maintaining a high local concentration of drug at the targeted tissues. Therefore, the transportation capability of hydrophobic/hydrophilic molecules is very important for a hydrogel-based drug delivery system. The drug diffusion and release behavior through the polymer network can easily be regulated by manipulating the crosslinking density in the gel matrix and thus controlling the porosity (mesh size) of hydrogels [1]. For their use as scaffolds for tissue regeneration or as tissue adhesives in surgical reparation, the bioadhesion ability of hydrogels is also a significant property that allows tissues and cells to adhere to hydrogels. The hydrogels can be modified by using linker molecules that enable non-covalent or covalent molecular interactions between the gel matrix and its surroundings to improve the cell/tissue-adhesive properties [4,5]. The design of hydrogels with a good mechanical performance is of crucial importance in many biomedical application areas. In addition, the water content of a swollen hydrogel plays a key role in the biomedical applications of hydrogels, as it can significantly influence the solute diffusion and mechanical properties of the hydrogels [5,6]. In sum, the suitability and performance of hydrogels as unique materials in a biomedical application largely depend on their network architecture. The network structure of hydrogels can be characterized mainly by analyzing the polymer volume fraction in the swollen state, detecting the polymer chain length between two adjacent crosslinking points and calculating the corresponding mesh size [7,17]. The molecular structure of hydrogels can also be tuned by the selection of polymer, crosslinker and type of crosslinking as well as by the control of the crosslinking density, which offer great versatility in preparing hydrogels with tailorable network and mechanical strengths.

As mentioned above, due to the aforementioned remarkable properties, hydrogels as promising materials are highly suitable for different applications, especially for diagnostic and therapeutic applications in biomedical areas. They not only can serve as a vehicle to load and deliver drug or protein to tissues [1], but also can act as scaffolding materials to fabricate artificial tissues and organs for transplantation and serve as wound dressings, barriers or adhesives between material and tissue surfaces [4,7,8]. In addition, they are able to be applied to mimic the natural extracellular matrix (ECM) and thus offer support for cell migration, proliferation and adhesion, which can offer not only a solution for large-scale cell manufacturing and protein production, but also a platform for stem cell research and cell therapy [5]. Moreover, hydrogels can be cast into practically any shape, size, or form. And especially the stimuli-responsive hydrogels can respond to their surrounding environments, which have opened a new avenue for biomedical research in numerous leading edge applications, such as medical and biological sensors and actuators, diagnostic imaging and devices [3,5,7].

Overall, hydrogels have become a popular choice of materials for biomedical and pharmaceutical applications. However, despite the many favorable characteristics of hydrogels, problems still exist in many aspects, limiting their broad clinical applications. For example, for the hydrogel-based drug delivery system, the homogeneity and quantity of drug loading into hydrogels is often very difficult, particularly in the case of hydrophobic drugs [1]. The large pore sizes and high water content of most hydrogels often lead to relatively rapid drug release [1]. The premature drug release outside the targeted site not only decreases the therapeutic efficacy, but also causes serious local or systemic toxicity [18]. Besides, some hydrophobic drug crystal precipitation tends to occur in the hydrogels, generally causing poor formulation stability and a low drug-loading capacity [19]. Hydrogels are generally prepared from a single polymer network, which often does not hold all the required mechanical and biological properties for tissue engineering applications. They commonly tend to be too flexible to meet the mechanical demands of certain applications, leading to premature dissolution or flow away of the hydrogels from a targeted local site [1,3,6,7]. Moreover, the current hydrogels used in tissue engineering applications are still unable to replicate the complexities of the native environment, and thus often leading to unsatisfactory results. There is an increasing need for functional hydrogels to be used as biosensors, actuators and biologic devices, which are generally based on the measurement of hydrogels’ changes in response to magnetic, optical and/or electrical stimuli. However, most of hydrogels made from polymers are completely inert and non-responsive under the magnetic and electrical conditions [3,7]. Apparently, each of these issues significantly restricts the practical use of hydrogels in the clinic. As a result, it is imperative to develop new materials and novel strategies for the introduction of physicochemical, mechanical, and biological functionality into hydrogels to meet the rigorous and diverse demands.

One of the efficient and simple approaches to improve the hydrogels properties and to include multiple functionalities is the incorporation of different entities into hydrogels to fabricate composite hydrogel matrices [3,20,21,22]. For example, the strategy of introducing the secondary polymers into hydrogels by physical blending or copolymerization to form hybrid hydrogels or interpenetrating network structure (IPN) hydrogels [3]. A variety of nanoparticles (NPs) have distinct physicochemical properties and favorable functions that are not commonly found in polymers. Recently, considerable efforts have been made to incorporate various kinds of NPs into gel matrices to create novel nanocomposite hydrogels with superior properties and tailored functionalities [20,22,23,24,25,26,27]. This review will summarize the most recent developments in the domain of nanocomposite hydrogels in biomedical and pharmaceutical applications, specifically highlighting some interesting discoveries in composites of hydrogel and polymeric NPs for biomedical applications.

## 2. Nanoparticles and Their Biomedical and Pharmaceutical Applications

**Figure 2:**
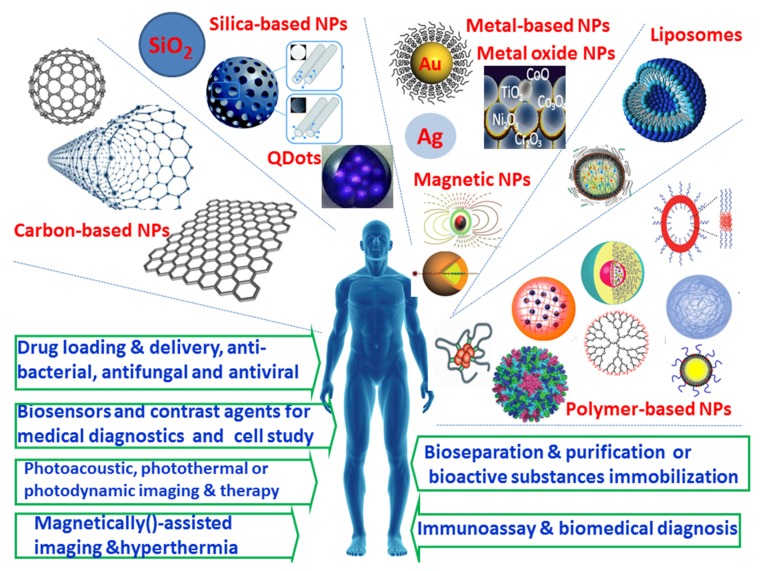
Examples of typical nanoparticles and their applications in biomedical fields.

Parallel to the aforementioned developments in hydrogels, nanoparticulate systems have gained a great amount of attention in the past several decades as one of the most promising biomedical materials, due to their unique physicochemical properties, nano-sized characteristics, controlled shape and versatile modification possibilities as well as well-defined multifunctionalities [28,29,30,31,32,33,34]. Significant progress has been achieved in preparation and functionalization of various nanoparticles for a diverse array of biomedical applications. Researchers have developed and employed a wide variety of nanomaterials, such as carbon-based NPs [35,36] (e.g., carbon nanotube, graphene, fullerene), silica-based NPs [37,38], and other inorganic NPs [30,39], semiconductors NPs [40,41], metal and metal oxide NPs [33,42] (e.g., Au [43,44,45], Ag [46] and iron oxides [47,48]), as well as liposomes [49,50,51], and polymer-based NPs (e.g., polymeric NPs, micelles and nanogels, polymersomes, dendrimer, polymer-drug conjugations, emulsion particles and protein NPs) [28,32,34]. As shown in Figure 2, these nanomaterials provide a powerful platform for site-specific and controllable delivery of drugs, gene, proteins and other bioactive agents; some of them exhibited noticeable antibacterial, antiviral and antifungal activities. Some other inorganic nanoparticles, such as gold NPs, semiconductor NPs and magnetic NPs, can be used for photoacoustic, photothermal or photodynamic as well as hyperthermal therapy, due to their unique physical properties. In addition, some functional NPs can find applications for a new generation of intelligent biosensing, bioseparation, bioimaging, cell labeling and diagnosing as well as monitoring of cells and tissues.

NPs enter the human body by injection, inhalation, ingestion or through the skin. Once invading a biological milieu, NPs will inevitably contact with a huge variety of biomolecules in body fluids or blood, such as sugars, proteins, and lipids, which could immediately coat the NP surfaces leading to the formation of the so-called “protein corona” [52,53,54]. If the NPs can avoid the clearance by the reticuloendothelial system (RES) and/or mononuclear phagocytic system (MPS) and remain in circulation, they will reach the target tissues or cells and finally be internalized by targeted cells. Undoubtedly, NPs’ transport behavior in blood, uptake and clearance by macrophages, consequent biodistribution and metabolism, as well as their internalization by targeted cells are critical for their therapeutic and diagnostic applications and are dictated by the physicochemical properties of NPs, such as hydrophilic–lipophilic property, surface feature and particle size, particle shape [52,53,54,55,56,57,58].

Generally, the biological use of NPs requires hydrophilicity for good dispersion in water or serum to impede aggregation and opsonization, and thus NPs are usually coated with hydrophilic polymers, surfactants, and biodegradable copolymers, e.g., PEG. However, hydrophobicity is also required to promote the interaction of NPs with the cellular membrane to improve uptake into cells. As a result, the anchoring of the hydrophilic ligands to the NPs surface can prolong the circulation time of NPs, but they also can dramatically suppress the interactions between NPs and cells, thus leading to a minimal internalization [32,45,53,54,59]. It was also worth pointing out that both corona formations between NPs with proteins in body fluids and interactions between NPs-bound ligands with cell membranes are influenced by the ligand type, ligand structure and ligand packing density on the surface of NPs.

Surface charge is another important factor that can definitely affect the fate of NPs administered in biological systems. Because the cell membranes have a slight negative charge and cellular uptakes are driven by electrostatic attractions, positively charged NPs, compared with NPs with a neutral or negative charge, have higher affinity with negatively charged cell membranes, leading to a faster and more efficient cellular uptake [53,60]. However, positive NPs always have serious aggregation and rapid clearance after injection due to the nonspecific interactions with blood components.

In addition of surface properties of NPs, nanomaterial size and shape also contribute significantly to their biodistribution in circulation and the clearance from the body, as well as interaction with subcellular organelles, cells, tissues, and organisms [54,55,61]. NPs, are often defined as solid particles with a size in the range of 10–1000 nm. However, the size of NPs suitable for systemic administration for therapeutic applications might be in the range between 2 and 200 nm [52,54,55,61]. Firstly, the size of NPs can affect their permeability out of the vasculature, the clearance from the body and biodistribution *in vivo*. Some previous studies have indicated that the smaller the size of NPs will lead to the less the interactions with endothelial cells and the higher the concentration in a blood vessel, as well as the longer the blood circulation time [53,54,62]. NPs with size of less than 20 nm showed wide spread in various organs by crossing the tight endothelial junctions. They were found in almost all organs, including the blood, heart, lungs liver, kidneys, spleen, testes, thymus, and brain. In general, small particles are less likely to be taken up by macrophages than large ones. However, NPs with size of less than 20 nm are rapidly excreted through the glomeruli of kidney. Large NPs tended to aggregate under physiological conditions and be retained mechanically by capillaries, which were mainly detected only in the blood, liver and spleen [53,54,62]. Generally, the ideal size of NPs for long circulation should be in the range of 20–200 nm. The size of NPs should be larger than 20 nm in diameter in order to avoid filtration by the kidney and smaller than 200 nm to avoid specific sequestration by fenestra of liver and sinusoids in spleen. In the case of accumulation of NPs in tumor tissues, the pores or fenestrations in tumor tissues are much larger (>100 nm) than the tight endothelial junctions of normal vessels (<10 nm), allowing NPs to passively accumulate inside the tumor (termed enhanced permeation and retention (EPR) effect) after extravasation. Researches have demonstrated that the capacity of NPs to navigate between the tumor interstitium decreased with increasing size [58]. Large NPs are very difficult to extravasate far beyond the blood vessel, whereas small NPs easily enter the tumor parenchyme through tumor vasculatures, but they remain there only transiently due to the rapid secretion from the tumor. The size of NPs is also one of the major factors determining the efficiency and mechanism of cellular uptake. NPs are internalized into the cell through various pathways, such as pinocytosis and phagocytosis. Large particles are most likely to be engulfed by macropinocytosis. Internalization of NPs with a diameter of less than 200 nm involved a clathrin-mediated endocytosis process. With size increasing, the caveolae-mediated internalization becomes more predominant [53,54,55]. The shape of NPs also plays a very important role in their *in vivo* behavior and interaction with cells. Some studies suggested that spherical NPs of similar size were uptaken significantly more efficiently than non-spherical NPs, due to greater membrane wrapping time required for the elongated NPs. However, non-spherical NPs exhibit superior properties to their spherical counterparts in terms of escaping from phagocytosis, circulating in blood and binding to the target tissues [53,54,55,58,61]. In sum, prior to the widespread applications of NPs in biomedical and pharmaceutical field, it should be very important to demonstrate how the physicochemical properties of NPs interact with subcellular organelles, cells, tissues, and organisms. And the effect of physicochemical properties of NPs on biological systems should be fully taken into consideration toward the development of the next generation of therapeutic and diagnostic NPs.

Despite the many advantages making NPs a great candidate for various biomedical applications, there remains a lot of pressing challenges for us to face in the future. In certain applications such as the development of drug delivering systems, for example, “Soft” drug carriers (including liposomes, micelles, dendrimers and polymeric NPs) often suffer from the premature release of drugs under harsh environmental conditions and incontrollable drug release rate *in vivo*. These bioorganic NPs, such as liposomes and polymeric micelles, are generally formed by spontaneous aggregations, which are dynamically unstable system owing to the weak nature of the cohesive interactions. They often displayed low resistance to dilution-induced disassembly *in vivo* and high instability during storage as well as relatively small loading capacity [63]. Compared to the above soft organic NPs, “Hard” nanomaterials (e.g., NPs originated from carbon, silica, metal and metal oxides, quantum dots (QDots), *et al.*) have many fundamental advantages. These various inorganic NPs possess plenty of intrinsic functionalities, because of the optical, magnetic, electrical and physical properties. They could be made into integrated and multifunctional systems, including stimuli-responsive release, specific tissue/cell type targeting, *in vivo* imaging for diagnosis, photothermal treatment, and drug delivery monitoring, which are normally unable to be found in the above “Soft” nanomaterials [30,35,43,46,47]. Besides, “Hard” inorganic NPs are generally consisted of a three-dimensional arrangement with linked atoms mainly via covalent or metallic bonds. And therefore these particles are highly ordered and extremely stable in circulation and during storage [30,35,43,46,47]. However, compared with the polymeric NPs, these inorganic NPs often suffer from a very low biocompatibility and a very high foreign body response or inflammatory response [39,43,45,59]. In fact, as for the biomedical applications of most of nanomaterials, the toxicity toward normal tissues or non-targeted cells is the most formidable obstacle to be overcome. In addition, to exert its pharmacological effects, NPs administrated must be remained in the circulation long enough and reach the required concentration. Therefore, another main challenge toward designing NPs for biomedical applications is how to avoid rapid clearance from circulation by MPS or RES after administration. It was well known that, once entering blood circulation, NPs will be rapidly coated with serum proteins and then cleared through renal systems or sequestered in the liver or spleen [53,54,58,62]. In order to enhance the biocompatibility and evade the opsonization process, various biomolecules and ligands were anchored by physical adsorption or chemisorption on the surface of NPs, especially for inorganic NPs. The functionalized NPs displayed higher colloidal stability, improved biocompatibility as well as anti-opsonization activity under physiological conditions [45,59]. However, the physicochemical characteristics of the particle can drastically change with even the slightest changes in the morphology and surface of NPs arising from functionalization process, significantly impairing their functionality and performance the high cost. Moreover, the complicated synthesis steps and low stability as well as harsh reaction condition and tedious purification process limit their large-scale production. Therefore, there is still an urgent need to develop a couple of simple and efficient strategies to functionalize NPs in order to foster the successful applications of NPs for diagnostic and therapeutic purposes.

## 3. Composites of Hydrogels and Nanoparticles

### 3.1. Nanocomposite Hydrogels: A Brief Overview

As mentioned above, because of their unique structures and properties, the three-dimensional hydrogels and NPs have shown a very high potential for medical therapeutic and diagnostic applications. However, some inherent limitations of hydrogels and NPs inhibited their widespread applications. For example, with the rapid development of biomedicine, there is an increasing demand for multifunctional hydrogel with enhanced mechanical property. However, the limited functionalities and inferior mechanical properties of hydrogels have hindered their wide applications. As for NPs, they have plenty of unique multifunctionalities for biomedical applications, but the high instability, low biocompatibility and opsonization activity still remain significant clinical challenges. To meet increasingly rigorous requirements for biomedical use, various strategies have been explored to surmount the drawbacks but meanwhile maintain the advantages of hydrogels and NPs. Among them, the design and fabrication of novel biomaterials with desirable functions and excellent performances by innovative combination of these two entirely different types of materials has gained enormous attention [20,22,23,24,25,26,27,64].

**Figure 3:**
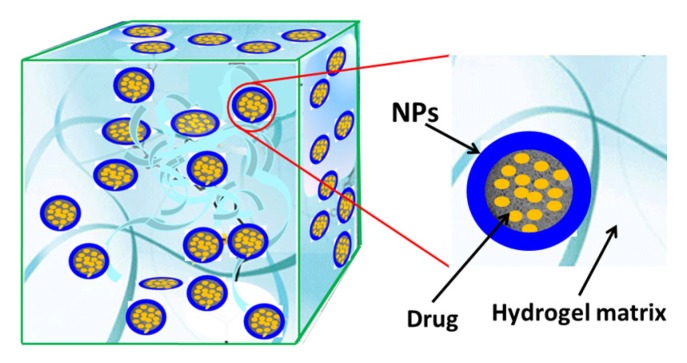
Schematic illustration of typical nanocomposite hydrogels from hydrogels and drug-loaded nanoparticles (NPs). NPs were non-covalently or covalently immobilized in a hydrogel matrix.

Composite materials are materials which consisted of two or more constituent materials with significantly different physicochemical properties. When two or more components are combined in a single entity where the individual components remain distinct and separate, the resultant composite materials can exhibit markedly different characteristics from the individual components. Originally, the advantages of fabrication of composite materials by incorporating a second component into the main matrix are usually for the purpose of strength enhancement and reinforcement. For example, mud and straw were combined to form bricks several thousand years ago. Concretes are actually a composite of stones in a matrix of cement, which have been widely used for buildings. Recently, to address the specific biological and clinical challenges, the incorporation of nanostructured fillers into hydrogels has been developed as a popular means for the creation of novel materials with diverse functionality. The resulting nanocomposite hydrogels, also known as hybrid hydrogels, can be defined as composite material that physically or covalently incorporates nanosized particles or nanostructures into a matrix of cross-linked polymer networks. These nanocomposite hydrogels have found fascinating interest for various biomedical applications due to combining the characteristics of a nanoparticulate material (e.g., very small size and high surface area) with hydrogel system (e.g., hydrophilicity and extremely high water content) [64]. As shown in Figure 3, the rigid NPs and flexible polymers can be looked as bricks and mortar. The polymer mortar connects the bricks to maintain the structural integrity and offer stability to the NPs; meanwhile NPs bricks render new functionalities to the composites. As a result, the challenges in the application of hydrogels and NPs, as mentioned above, could potentially be overcome by the fabrication of nanocomposite hydrogels. For example, the main aim of incorporation of NPs into hydrogel networks in the early attempts was to improve mechanical properties of hydrogel [65,66]. Usuki *et al.* significantly improved the tensile strength of hydrogels by incorporating montmorillonite, a type of natural silicate mineral (“clay”) NPs, into nylon-6 network [65]. The inorganic clay NPs are also rendered hydrophilicity and solubility when they are dispersed into 3D polymer networks, which can meet the essential requirements for the reasonable wound dressing with some desirable characteristics [67]. In recent reports, nanocomposite hydrogels prepared by incorporation of silica NPs with hydrogels showed remarkable improvements in mechanical stiffness, tissue stickiness and bioactivity compared with unmodified hydrogels [68,69]. In addition, the incorporation of metal NPs into hydrogel networks will impart superior physicochemical properties absent in the individual components. For example, incorporation of gold or silver NPs into a polymer matrix will render excellent antimicrobial property [70,71]. Similarly, the entrapment of magnetic NPs into polymer networks can make hydrogels exhibit magnetic character. Meanwhile, the NPs embedded in hydrogel matrix are endowed with particular surface properties, which can avoid aggregation and sedimentation [72]. In sum, the innovative combinations of hydrogels and NPs generate not only structural diversity but also a plurality of property enhancements [64].

**Figure 4:**
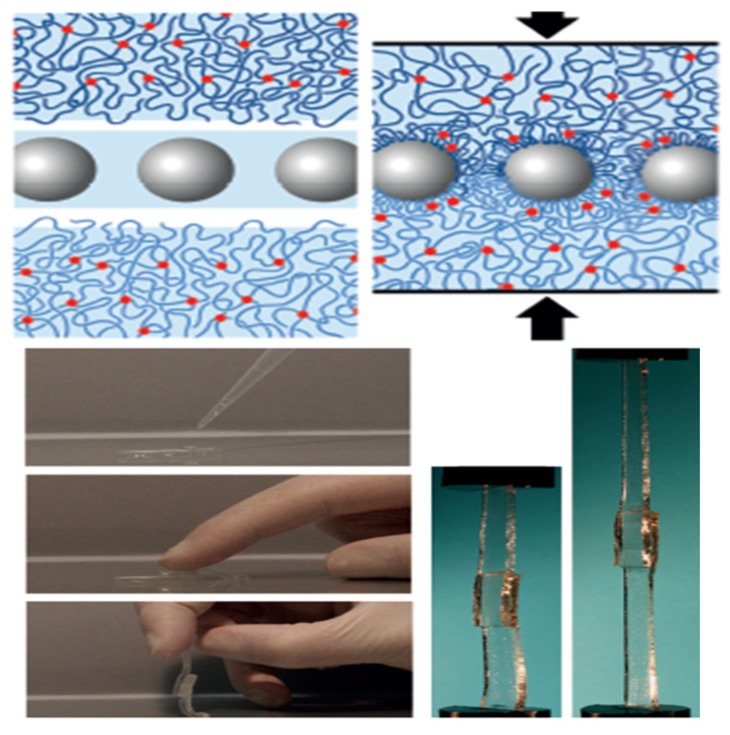
Schematic illustration of the concept of gluing swollen polymer networks together using silica NPs, reproduced with permission from [81]. Copyright Nature Publishing Group, 2014.

Apparently, the fabrication of composite materials of hydrogels and NPs provides an efficient route to improve their performances and expand their application scopes. A range of NPs such as carbon-based nanomaterials (carbon nanotubes (CNTs), graphene, fullerene), inorganic/ceramic NPs (silica, silicates, clay, hydroxyapatite, calcium phosphate, quantum dots (QDots)), metal/metal-oxide nanoparticles (gold, silver, copper, platinum, iron oxides and titanium oxides) and organic NPs (polymer NPs, micelles, polymersomes, dendrimers and liposomes), have been integrated within the hydrogel networks to obtain nanocomposite hydrogels with tailored functionality and superior physicochemical and biological properties. As the definition suggested, nanocomposite hydrogels can be made through the combination of NPs and hydrogels via various mechanisms. Recently, Thoniyot *et al.* [27] summarized the main approaches to prepare nanoparticle-hydrogel composites. The simplest method to prepare a nanocomposite hydrogel is the gelation of a suspension of pre-ready NPs in a solution of hydrogel-forming monomer. For example, Sershen *et al.* report a gold (Au) NPs- acrylamide hydrogel composites can be prepared by adding pre-formed Au NPs into a solution of acrylamide monomers and then initiating the polymerization by addition of the gelation initiator [73]. This approach has been widely used to prepare a variety of nanocomposite hydrogels containing various NPs. However, some drawbacks limit its wide applications, such as the aggregation of NPs in monomer solution before and during the gelation process, and the leaching of NPs out of the hydrogel matrix if the cross link density is low [74,75]. Another common approach to obtain nanocomposite hydrogels is to physically introduce NPs into a hydrogel networks after gelation. Some hydrogels, such as PAM hydrogel can highly swell in water but dramatically shrink in an aprotic solvent acetone. NPs can be introduced into this kind of hydrogels in the repeated swelling-shrinking process [76,77]. Typically, the suspended NPs in water will be embedded in the swelling hydrogel when the shrunken gel in acetone was placed in an aqueous solution of NPs. After washing out the weakly surface-adsorbed NPs, the nanocomposite hydrogel was obtained [76,77]. The NPs can be introduced into hydrogel matrix and remain attached within the gel, possibly owing to the physical entanglement and/or hydrogen bonding interactions between the polymer chains and NPs surface. Alternatively, the incorporation of NPs into pre-formed gels could also be achieved by repeated heating, centrifugation and redispersion according to similar mechanism [78]. The NPs can also be created *in situ* after gelation of the hydrogel and subsequent conversion of the nanoparticle precursors suspended in the gel [79]. In fact, the porous structures of hydrogel networks are very suitable for *in situ* formation of NPs, as the free space among the gel network provided a nanoscopic pot for formation and growth of NPs without aggregations [20,27]. Apparently, this method involves loading NPs precursors into the hydrogel networks, rather than preformed NPs. In a typical procedure, Au (III) or Ag (I) ions were embedded PNIPAM or PAA hydrogel networks and then the Au or Ag ions functionalized polymer hydrogels were reduced or hydrolyzed to yield metal NPs-hydrogel composites [79]. One particularly innovative approach to yield NPs-hydrogel composites is the use of crosslinker groups on the outer surface of NPs to form the hydrogel matrix [80,81]. This concept was well demonstrated by Rose *et al.* for adhesion between two hydrogels [81]. A rapid and strong adhesion between two hydrogels can be achieved by adding a droplet at room temperature of silica NPs solution on the surface of the hydrogels, as shown in Figure 4. This method was dependent on the ability of NPs to act as a connector between polymer chains or to be adsorbed onto the polymer gels. Finally, the nanocomposite material can also be obtained by using the interactions among NPs, polymers, and distinct gelator molecules to form positively charged polymers into NPs-hydrogels composites [82]. These strategies for the fabrication of nanocomposite hydrogels bearing tailored functionality have opened up new possibilities in fabricating advanced biomaterials for various biotechnological and biomedical and applications. Different types of NPs-hydrogel composites and their associated properties and applications will be described below.

### 3.2. Nanocomposite Hydrogels from Hydrogels and Carbon-based NPs

**Figure 5:**
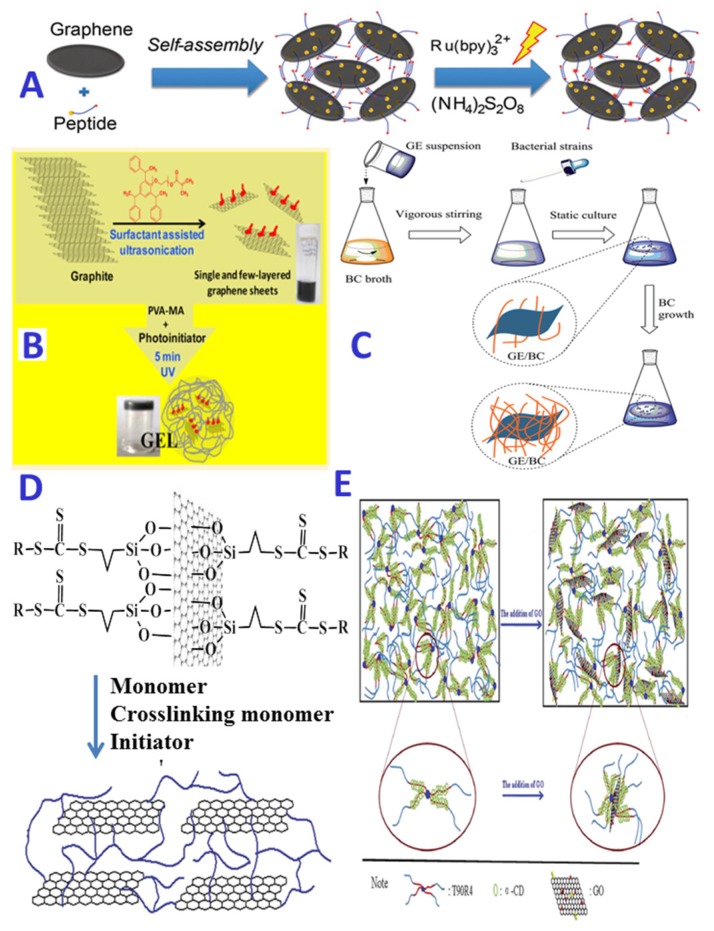
Schematic illustrations of incorporating graphene-based materials into hydrogel matrixes; The formation of graphene-hydrogel composite materials by (**A**): self-assembly and photo-crosslinking between graphene and peptide, reproduced with permission from [99]. Copyright Royal Society of Chemistry, 2015; (**B**): photopolymerization of vinyl moieties present on surface of graphene and polymer chains, reproduced with permission from [91]. Copyright American Chemical Society, 2015; (**C**): *in situ* biosynthesis, reproduced with permission from [103]. Copyright Royal Society of Chemistry, 2014; (**D**): surface-grafted polymerization, reproduced with permission from [104]. Copyright American Chemical Society, 2012; (**E**): supramolecular self-assembly by hydrogen bonding, reproduced with permission from [102]. Copyright Royal Society of Chemistry, 2015.

Hydrogels, generally prepared from a single or multiply polymer networks, often lack all the required mechanical properties for tissue engineering applications. And they are also short of functions (such as electrical, optical and thermal conductivity) for the applications of controlled drug delivery or biosensing and bioimaging. Therefore, some strategies have been employed to improve the properties of hydrogels, mainly by inclusion different entities into hydrogel matrices to create composite hydrogels [3,24,25,27]. In the past two decades, carbon-based NPs, such as carbon nanotubes (CNTs), graphene and fullerene (C_60_) have gained worldwide popularity, due to their excellent multifunctional feature and unique physicochemical properties (*i.e.*, mechanical strength, corrosion resistance, low cost and friendly to the environment, especially thermal, electrical, and optical conductivity). As a result, extensive research efforts are reported to utilize the favorable and unique properties of carbon-based NPs to create composite materials for various applications, including sensors and actuators, photothermal and photodynamic therapy, diagnostic imaging and drug delivery devices [36,83,84]. For example, due to their remarkable mechanical properties, carbon-based NPs have been widely used to incorporate into hydrogels to improve their physical and mechanical properties for a variety of tissue applications [85,86,87,88,89,90,91,92,93,94]. A great number of studies have demonstrated that a small amount of carbon-based NPs, such as CNTs or graphene, in the polymer matrix can markedly change its mechanical, electrical and thermal properties [87,88,89,90]. However, the key problem of the practical use of CNTs is their poor dispersibility in water, organic solvents and polymer matrix [87,88,89,90,95,96]. Generally, some pretreatments, such as functionalization with strong acid or surface modification with surfactants, polymers or proteins, are needed to improve the dispersion of CNTs [25]. Among various carbon-based NPs, graphene-based materials (including graphene, graphene oxide (GO) and reduced graphene oxide (rGO)) have been extensively explored as a novel reinforcing agent to support polymers for biomedical applications, due to their distinctive properties, such as large surface area (2620 m^2^/g), high aspect ratio, outstanding mechanical properties (Young’s modulus of 1 TPa and intrinsic strength of 130 GPa), excellent thermal and electrical conductivity as well as good optical transparency [36,84,97,98]. However, graphene is neither lipophilic nor hydrophilic, often causing a high difficulty in their processing and low compatibility with polymer matrices [97]. In contrast, GO, a single monomolecular layer of graphite with various oxygen containing groups such as epoxide, carbonyl, carboxyl and hydroxyl groups, is amphiphilic and thus can be dispersed in water and alcohol through hydrogen bonding. This will significantly facilitate molecular dispersion of GO in polymeric matrix and simplify the processing of GO-based composites. As a result, GO as a reinforcement agent can be directly introduced to into polymer matrices by solvent blending method. Nevertheless, as shown in Figure 5, the stable inclusion of graphene in a polymer matrix often requires pretreating graphene (such as surface and structural modifications) or post-treating hybrid hydrogel (such as crosslinking) [91,97,99]. Undoubtedly, the incorporation of graphene-based materials into hydrogel has proved extremely valuable for the improvement of the mechanical and thermal properties of the hydrogels. However, it was worth pointing out that the embedment of graphene-based materials into a hydrogel matrix is often indispensable, especially when these graphene-based materials were used in biomedical and biological fields. Normally, the graphene sheets have sharp edges, which can assist penetration through cell membranes. However, the sharp edges will also markedly enhance the cytotoxicity [100]. The confinement of graphene in a water-rich environment of hydrogels can change its interaction with cells and thus offer a solution to prevent this effect [91].

In addition, hydrogels commonly made from non-conductive polymers are not conductive towards optical, thermal and electrical stimuli. However, there is an increasing need for conductive hydrogels in the biomedical applications, such as biosensors, actuators and neural tissue engineering. Therefore, carbon-based NPs with excellent optical, thermal and electrical conductivity are considered as highly promising materials to engineer conductive stimuli-responsive hydrogels [3,83,84,95]. A variety of optical or electrical-responsive nanocomposite hydrogels as actuator, biosensor or tissue engineering scaffold have been developed by incorporating with carbon-based NPs [91,92,101,102]. Overall, due to the fact that the carbon-based NPs often possess highly desirable functions that cannot be commonly found in polymers, the strategies of integrating carbon-based NPs into polymer hydrogels to develop composite hydrogels with enhanced mechanical strength and specialized properties (e.g., electrical, thermal and optical conductivity) are expected to be extensively explored for biomedical applications. And these and related topics have been well covered in several excellent reviews [3,85,95,97]. And therefore, as shown in Table 1, we just summarized the latest advances of experimental research on nanocomposite hydrogels from hydrogels and carbon-based NPs for biomedical and pharmaceutical applicants.

**Table 1:** Summary of the most recent advances of nanocomposite hydrogels from hydrogels and carbon-based NPs for biomedical applications.

Fillers	Hydrogels	Applications and Functions	Ref.
Carbon Nanotubes (CNTs)	PVA	Drug delivery; Incorporation of CNTs caused a significant increase in tensile strength, decrease in elongation at break, and increase in Young’s modulus of the PVA hydrogels; Improved the electrical conductivity and antibacterial activities of the hydrogel systems; CNTs imparted to hydrogels the ability of electrical stimulation-controlled drug release	[105,106]
	PHEMA	Tissue engineering; CNTs were added to improve electrical conductivity and mechanical properties; CNTs makes hydrogel more suitable as nerve conduits	[87]
	PNIPAM	Tissue engineering; Incorporation of CNTs could improve the bioactivities, especially supportive adhesion of PNIPAM to encapsulated cells and favor their efficacy in myocardial repair	[88]
	PAM	Biomaterials; CNTs can significantly improve the mechanical properties and swelling behavior of PAM hydrogels	[89]
	P(AM-co-EBA)	Drug delivery; CNTs was covalently included into films and acted as a functional element for both improving the electro-responsivity and modulating the release of anionic and cationic drugs	[90]
	P(EGDMA-co-HEMA)	Tissue engineering; CNTs can improve the swelling and mechanical properties	[107]
	P(VPB-co-DMA)	Biomaterials; CNTs could increase storage moduli of the hydrogel and endow the hydrogel with self-healing property	[96]
	MPEG/α-CD	Drug delivery; CNTs improved the hydrophobic drug loading amount; MPEG not only dispersed CNTs in aqueous solution, but also functioned as hydrogel matrix by interacting with α-CD	[108]
	GG-g-AA	Drug delivery; CNTs can increase the hydrophobicity of hydrogel, ensure the high environmental stability and drug retention properties of hydrogel	[109]
	BC/SA	Drug delivery; CNTs can be used to obtain the composite hydrogel with the highest electric sensitivity; Drug release from hybrid hydrogels showed a significant pulsatile characteristic	[110]
	DNA	Tissue engineering; Incorporation of CNTs significantly strengthens the mechanical properties of DNA hydrogel; DNA improved the biocompatibility of CNTs and the resulting hybrid hydrogel	[111]
	PEI-Fc	Drug delivery; CNTs can improve the electrical sensitivity properties of hydrogel and can be used to tune the stiffness and degree of swelling	[112]
	GEL	Tissue engineering; CNTs supported the contractile property of engineered cardiac tissues, improved the formation of gap junction and enhanced the excitation-contraction coupling	[113]
	CS	Antimicrobial biomaterials; The addition of CNTs in the composite hydrogel scaffold significantly reduced the water uptake ability, increased the antimicrobial activity and decreased the toxicity	[114]
	COL	Tissue engineering; CNTs enhanced the proliferative potential of the cells; Hydrogel offers excellent 3-D conditions for the growth of cells; CNTs within the hydrogel significantly stimulate cell secretion of neurotrophic factors and expression of neural markers	[115]
	Ambidextrous gelators	Biomaterials; The inclusion of SWNTs within the ambidextrous gels improved the mechanical rigidity of the resulting soft nanocomposites	[116]
	Peptide	Tissue engineering; Peptide enables the dispersion of the CNTs in aqueous media; CNTs make hybrid hydrogels have superior physical and mechanical properties	[117]
	Lysozyme	Drug delivery and tissue engineering; CNTs contributed to gelation, improved mechanical properties and improved the biological activity of hydrogels	[118]
Graphene	PAA	Drug delivery; Graphene/PAA hydrogel was prepared with pH-sensitive, good thermal, strong mechanical and excellent elastic properties	[119]
	PMAA	Drug delivery; The graphene hybrid hydrogels with enhanced mechanical, electrical, and thermal properties were prepared by *in situ* free radical polymerization, which exhibited controlled drug release in a pulsatile fashion upon the ON/OFF application of low electrical voltages	[120]
	PVA/P(MA-co-NIPAM)	Drug delivery; Small amounts of embedded graphene affect the behavior of mechanical and thermal properties of the hydrogel without altering its hydration state; The entrapment of graphene increase the possibility for functionalization and its dispersibility in water	[91]
	Bacterial cellulose	Tissue engineering; Graphene was used to reinforce various hydrogels, leading to excellent mechanical, electrical and biological properties; Graphene also changed the crystallinity of bacterial cellulose	[103]
	CS/GEL	Neural tissue engineering; Graphene improved the electrical conductivity and mechanical properties, but decreased the porosity, swelling ratio and *in vitro* biodegradability	[92]
	β-CD /PDMA	Drug delivery; β-CD endowed the self-healing property of hydrogel as anticancer drug carrier, more drug loading and better drug release behavior	[121]
Graphene Oxide (GO) & reduced graphene oxide ( rGO)	PEG	Drug delivery; PEG endowed GO high aqueous solubility and stability in physiological solutions; GO imparted to hydrogel superior loading capacity for hydrophobic drug	[93]
	PLA–PEG–PLA	Drug delivery; Hydrogel can serve as a sustained delivery depot of the drugs. GO can increase drug loading capacity	[94]
	PEGDGE	Drug delivery; Enhanced mechanical and electrical properties were observed with increased rGO content; Hydrogel can greatly improve the stability of rGO dispersions	[101]
	PAA/GEL	Tissue engineering; GO worked as reinforcement agent can significantly increase the tensile strength (71%) and elongation at break (26%) of composite hydrogels	[122]
	GEL	Drug delivery; GO worked as physical crosslinker, and the multiple crosslinking sites between gelatin chain and GO nanosheet rendered GO–gelatin hybrid hydrogels high mechanical performance; Drug release behaviors from these hydrogels could be manipulated by controlling the crosslinking density and pore sizes of the hydrogels	[123]
	α-CD/PEO-PPO	Drug delivery; The incorporation of GO enhanced the mechanical strength and stability of the hybrid hydrogels enormously; Hybrid hydrogels can really exhibit a sustained drug release effect	[102,124]
	Peptide	Drug delivery; Mechanical stability, syringe-injectability, low erosion rate, and near-infrared light triggered release functionalities	[99]
	KGM/SA	Drug delivery; GO as a drug-binding effector for anticancer drug loading and release; SA as the pH sensitive agent	[125]
	CS	Blood toxins adsorption; The incorporation of graphene oxide into the chitosan matrix improved both the Young’s modulus and the compressive strength of the hybrid hydrogel, as well as its adsorption capacity for bilirubin	[126]
	clay-PDMA	Biomaterial dressing; GO sheets in the hybrid hydrogels acted as not only a collaborative cross-linking agent but also as a NIR absorber to absorb the NIR irradiation energy and transform it to thermal energy rapidly and efficiently, resulting in a rapid temperature increase of the GO containing gels.	[127]
Fullerene (C_60_)	PNIPAM	Drug delivery; The incorporation C_60_ improved the mechanical strength and decreased the swelling ratio and reduced the lower critical solution temperature of PNIPAM	[128]
	PHEMA	Drug delivery; Equilibrium swelling ratio is lower due to the presence of hydrophobic fullerene moieties; The glass transition temperature and thermo-stability were increased with increasing fullerene content	[129]
	GC-g-DMA	Tumor therapy; The incorporation C_60_ endowed the hydrogel an advanced property for endosomal pH targeting and an elevated photodynamic tumor cell ablation at pH 5.0 through an enhanced singlet oxygen generation at pH 5.0	[130]

Poly(acrylamide-co-*N,N*′-ethylene bisacrylamide) P(AM-co-EBA); Poly(methacrylic acid) PMAA; Glycol chitosan grafted with 2,3-dimethylmaleic acid (GC-g-DMA); Poly(*N,N*-dimethylacrylamide-4-((4-vinylbenzyloxy)carbonyl)phenylboronic acid) Poly(VPB-co-DMA); Polyethylene glycol monomethyl ether (PGME); Acrylic acid grafted guar gum (GG-g-AA); Bacterial cellulose and sodium alginate (BC/SA); Ferrocene-grafted polyethylenimine (PEI-Fc); Poly(methacryloyl-co-*N*-isopropylacrylammide) P(MA-co-NIPAM); Polylactide-poly(ethylene glycol)-polylactide (PLA-PEG-PLA); Konjac glucomannan/sodium alginate (KGM/SA); PDMAA: Poly(*N,N-*dimethylacrylamide) (PDMA); Glycol chitosan grafted with 2,3-dimethylmaleic acid (GC-g-DMA); Polyethylene glycol diglycidyl ether (PEGDGE).

### 3.3. Nanocomposite Hydrogels from Hydrogels and Inorganic NPs or Semiconductor NPs

**Figure 6:**
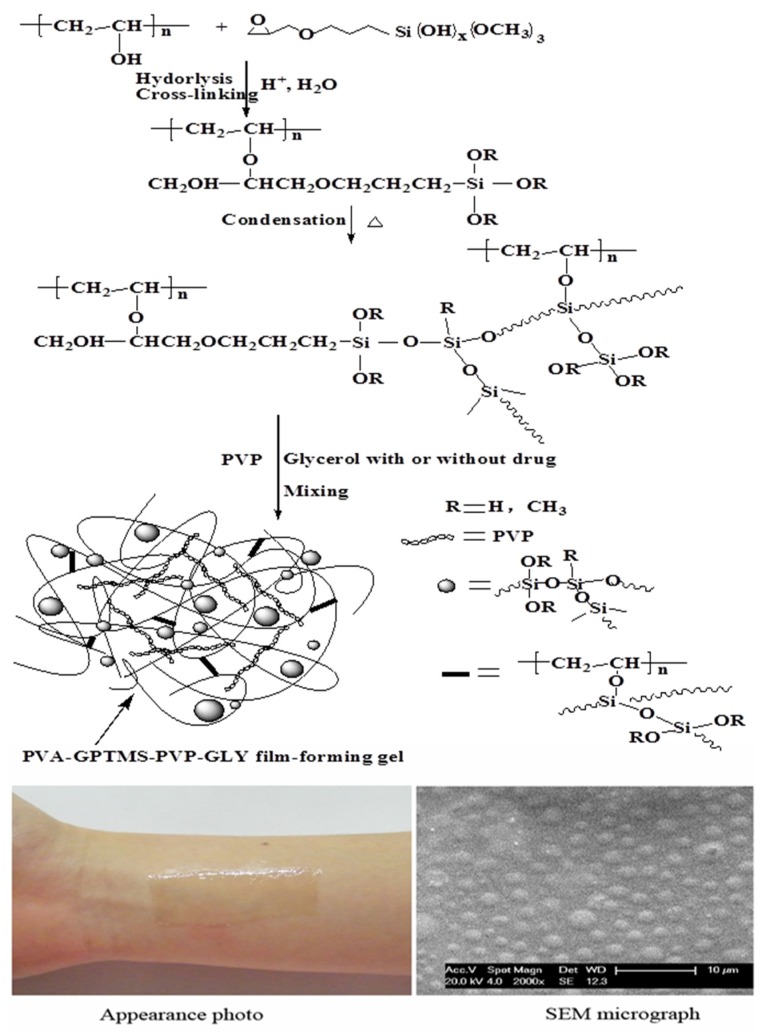
Illustration of the preparation of PVA-GPTMS-PVP-GLY film-forming gel and the appearance photo and SEM micrograph of the resultant gel, reproduced with permission from [69]. Copyright Elsevier, 2011.

In addition of carbon-based nanomaterials, some other inorganic NPs, such as silicon-based NPs and quantum dots (QDots) have been widely used to combine with the polymeric network to create composite materials with unique properties and functions. Especially, silicon-based NPs, including silica NPs (SiO_2_), mesoporous silica NPs (MSNs), hydroxyapatite (HAP) and bioactive glasses as well as various kinds of clay (such as montmorillonite (MMT), laponite (LAP), attapulgite and halloysite) are of greatest significance and have been utilized as the nanofillers in nanocomposite hydrogels, largely due to their excellent biocompatibility, chemical inertia, high thermal and mechanical stability, facile surface modification and easy functionalization. Most of these silicon-based NPs or nanoclays are already present in the body or are necessary for the normal functioning of human tissues [131]. Silicon is very important in skeletal development. Silicon promotes collagen type I synthesis and also stimulates the osteogenic differentiation in human stem cells [131]. HAP is a major mineral component and an essential ingredient of normal bone and teeth and HAP used biomaterials for bone regeneration can promote new bone ingrowth through osteoconduction mechanism without causing any local or systemic toxicity, inflammation or foreign body response [132,133]. Some studies demonstrated that some nanoclays, such as MMT and LAP, can induce osteogenic differentiation of human mesenchymal stem cells in the absence of exogenous growth factors [131,134]. It was well-known that many of the currently available polymer hydrogels suffer from poor mechanical properties and inability to induce mineralization. These unique bioactive properties and high mechanical strength of the silicon-based NPs and nanoclays will be of great interest to develop nanocomposite hydrogels with excellent mechanical and biological properties for the repair and regeneration of human tissues and body functions [3]. Especially, the anisotropic and plate-like, high aspect-ratio morphology of the nanoclays, lead to the formation of high non-covalent surface interactions between the nanoclays and polymer chains. These characteristics could therefore be used to create functional hybrid hydrogels via sol-gel process, which are very suitable for minimally invasive therapies, such as injectable tissue repair matrices or bone-related tissue engineering scaffolds [135,136]. In addition, due to their large specific surface area, controllable particle morphology and pore volume or interlayer space, excellent adsorption ability, ionic exchange capacity and outstanding adhesive ability, some silicon-based NPs, especially MSNs, MMT and LAP, have superb drug-carrying capability. They have been widely used to integrate into hydrogel drug delivery systems to increase drug-loading, manipulate drug pharmacological profiles, minimize drug degradation and decrease detrimental drug side effects [137,138,139]. We summarized the advances of nanocomposite hydrogels from hydrogels and silicon-based NPs for biomedical applications. As shown in Table 2, various kinds of silicon-based NPs have been incorporated in different synthetic and natural polymers via physical or chemical approaches to obtain bioactive nanocomposite hydrogels. These resulting hydrogels with enhanced mechanical properties, good biocompatibility and biodegradability, tunable cell and tissue adhesion, or improved drug loading and drug release profiles, have exhibited plenty of important applications in biomedical and pharmaceutical fields, such as injectable or implantable sustained drug delivery system, controlled cell and tissue adhesion surfaces, antimicrobial films or wound dressings, tissue engineering and cell based therapies. For example, in our previous study, a novel organic-inorganic silica NPs-based bioadhesive hydrogel as an alternative vehicle for transdermal drug delivery system was developed [69]. As shown in Figure 6, the direct formation of silica NPs in PVA hydrogel was observed due to the reaction between PVA and the hydrolyzed silanol of γ-(glycidyloxypropyl)trimethoxysilane (GPTMS) in the sol-gel process. The silica NPs dispersed in the matrix form hard segments in the hydrogel, which can not only significantly improve mechanical strength, skin adhesion properties and film-forming properties of PVA gels, but also can reduce the crystalline regions of PVA and hence facilitate the diffusion of drug and water vapor.

**Figure 7:**
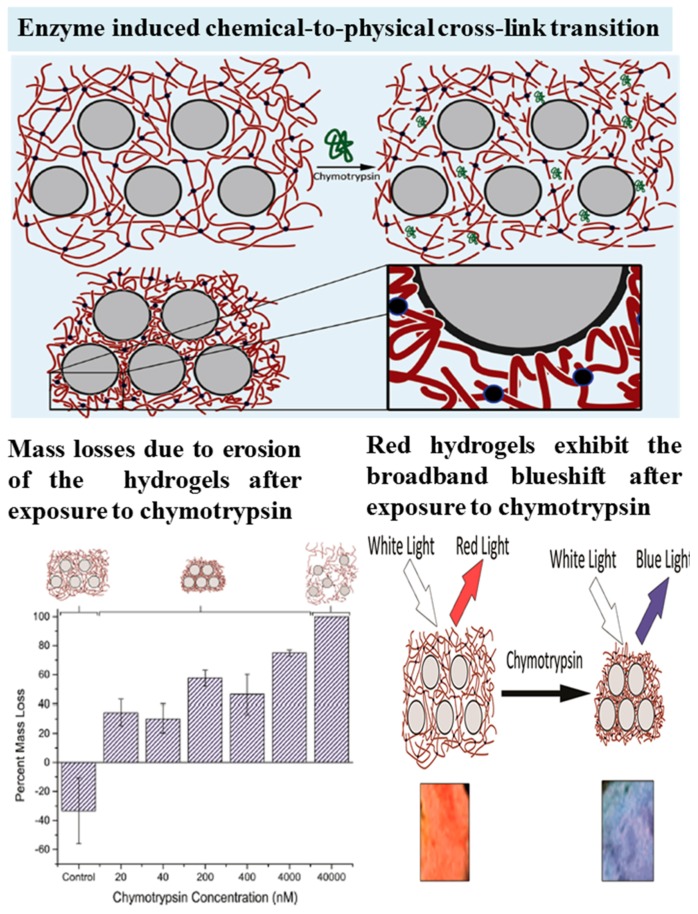
Depiction of the enzyme induced chemical-to-physical cross-link transition and subsequent mass loss and blue shift of nanocomposite hydrogels after exposure to chymotrypsin, reproduced with permission from [140]. Copyright American Chemical Society, 2015.

In another interesting study, Ayyub *et al.* [140] developed a nanocomposite hydrogel respond to proteolytic degradation with an increase in stiffness through the formation of physical cross-links. The nanocomposite hydrogel was consisted of a colloidal array of silica NPs distributed within a PEG-peptide hydrogel, as shown in Figure 7. The negatively charged silica NPs at a sufficiently high concentration within the hydrogel can form a regular periodic structure as a photonic bandgap to reflect visible wavelengths of light. The hybrid hydrogels would collapse in response to diverse biological stimuli, causing a 1200% increase in elastic storage and loss modulus. Moreover, the material provided threshold responses, requiring a certain extent of proteolytic activity before the transition occurred. This allowed for the fabrication of Boolean logic gates responding to a specific assortment of proteases. The study demonstrated that incorporating silica NPs at a high density within a polymer hydrogel can not only result in mechanically strong and tissue-adhesive nanocomposite hydrogels, but also fabricate soft materials with a unique and counterintuitive response to environment-stimuli. The protease responsive hydrogel nanocomposites reported in this study could offer avenues in degradation-stiffening and collapsing materials for a variety of biomaterial applications.

**Figure 8:**
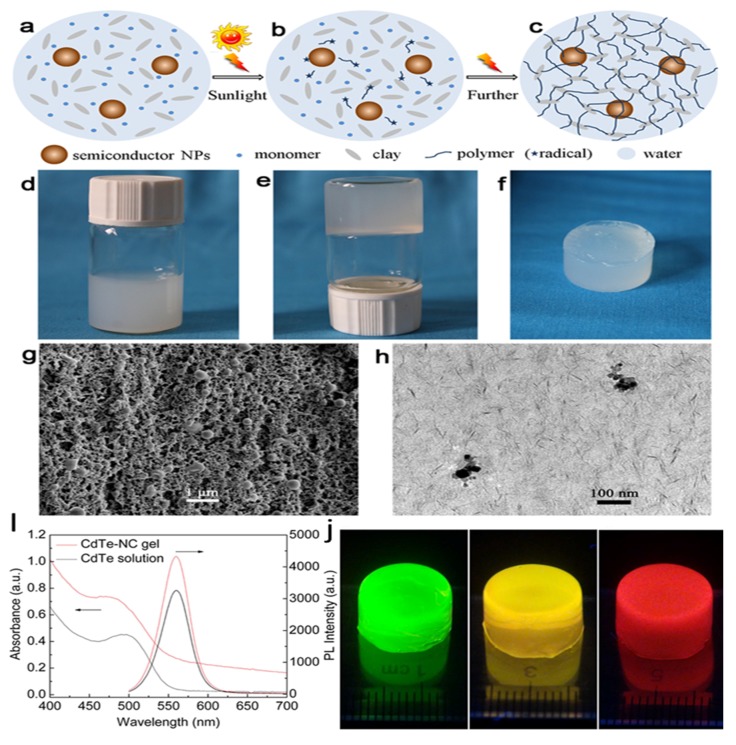
Proposed mechanism (**a**–**c**), photographs (**d**–**f**), and SEM image (**g**) and TEM image (**h**), UV-Vis absorbance and images under an ultraviolet lamp of the functional nanocomposite hydrogels prepared by a QDots-initiated polymerization approach under sunlight, reproduced with permission from [146]. Copyright Nature Publishing Group, 2013.

Among various inorganic NPs, semiconductor quantum dots (QDots), such as CdTe, CdS, carbon and silicon QDots, have attracted tremendous interest due to their unique photoluminescence behavior, including high quantum yield, large absorptivity, broad absorption spectrum, improved photo-stability, concentration and size-tunable emission [141,142,143,144,145,146,147]. These fluorescent QDots have been extensively studied and applied, such as imaging fixed and live cells all the way, high resolution localization of proteins and cellular structures, bioimaging *in vivo*, light-triggered drug delivery, immunoassays and innovative diagnostic methodologies [148,149]. For example, cells labeled with QDots with unique photoluminescence can be injected in small animals, which can be used to follow a particular pathway in the organism, such as the carcinogenesis process. However, due to high free energy of the surface and a large surface-volume ratio, QDots tend to easily aggregate in aqueous medium, resulting in fluorescence quenching. Therefore, the dispersity and stability of QDots are extremely important for their biomedical applications, such as biosensing, bioimaging, biolabeling and drug delivery. Many techniques, especially the surface functionalization by the attachment of ionic groups, polymers, lipids and proteins, have been employed to stabilize the colloidal QDs, but these approaches suffer from the complicated synthesis steps, harsh reaction condition and tedious purification process [29,141]. On the other hand, as described above, hydrogels have exhibited great potential in biomedical applications due to their unique physicochemical properties and porous network structures. In fact, hydrogels can also provide an ideal hydrated environment for the host of QDots. Moreover, the integration of fluorescent QDots into hydrogel matrix can not only provide protection to QDots against different harsh environments, but also render to the resulting composite hydrogels with unique photo-electronic properties that are highly suitable for therapeutic and diagnostic applications in biomedical fields [142,145,148,149,150,151,152]. Fabrication of fluorescent hydrogel by incorporating QDots in the polymer hydrogel matrix has achieved promising success. For example, QDots-loaded PNIPAM nanocomposite hydrogels have been used as light-controlled drug delivery system, thermo-sensitive biochemical monitor and sensor [144,150,153]. Generally, there are two distinct strategies for the incorporation of QDots into hydrogels, based on the covalent and noncovalent interactions between polymer chains and QDots. The former approach to QDs encapsulation involves the formation of covalent bonding, which is commonly realized by copolymerization or crosslinking of QDots carrying polymerizable ligands with proper monomers [141,146,154,155]. This strategy can guarantee the firm immobilization of QDots in the hydrogel matrix. However, this approach usually involves tedious polymerization or synthetic process, and usually requires that the hydrogel polymers are able to be chemically tailored. An alternative method involves encapsulating the QDots by the diffusion of the QDots into the preformed hydrogel or the mixture of the QDots with the precursor solution of hydrogel [147,156,157,158]. The entrapment processes are mainly dependent on the electrostatic interactions, hydrophobic interactions, hydrogen bonding, and host–guest interactions. These noncovalent bonding interactions, obviously, can drive the free QDots to be mostly inhaled into the hydrogels, due to the large surface area of hydrogels. However, for the case of physical embedding, the weak noncovalent interactions between the networks and the QDs may result in squeezing of the trapped QDs from the networks and a loss of luminescence [141,147,157]. A wide variety of polymer hydrogels, such as PAM, PEG, PVA, PVP, COL and AGL, have been used to entrap QDs into the hydrogel matrices by means of the above strategies. Typically, Liu *et al.* [141] combined a physical embedment and a subsequent covalent crosslinking to firmly encapsulate QDots into agarose hydrogels. Cysteamine-capped QDots can be entrapped into the pores of agarose hydrogels by hydrogen bonding between hydroxyl groups of agarose and the amino groups of cysteamine. Subsequently, oxalaldehyde and polyethylenimine were then incorporated to produce a covalently crosslinked network to further stabilize the encapsulation. In another study, Zhang *et al.* [146] developed a sunlight self-initiated polymerization approach to prepare multifunctional nanocomposite hydrogels by using QDots as the initiator and introducing clay as additional non-covalent crosslinking points, as shown in Figure 8. The resulting hydrogels exhibit excellent mechanical strength and high elasticity. Moreover, the self-immobilized NPs within the functional nanocomposite gels exhibit inherent physicochemical properties, such as large absorptivity, high photoluminescence and size-tunable emission, which are comparable to those of their original solution.

**Table 2:** Summary of nanocomposite hydrogels from hydrogels and silicon-based NPs for biomedical applications.

Fillers	Hydrogels	Applications and Functions	Ref.
Silica (SiO_2_) particles or NPs	HA/PNIPAM	Tissue engineering; SiO_2_ NPs improve mechanical properties; HA can improve biocompatibility; The stability and the rigidity increase with the amount of PNIPAM	[159]
	PNIPAM	Drug delivery and tissue engineering; SiO_2_ NPs improve the thermal and mechanical properties but decrease the swelling ratio	[160,161,162,163,164,165,166,167,168]
	PAM	Compression strength and elastic modulus of the composite hydrogels were significantly improved by adding SiO_2_ NPs compared with pure PAM hydrogels	[169,170,171,172,173]
	Silicone	Implantable medical devices; SiO_2_ can increase swelling behavior and improve stiffness of the hydrogel; Hybrid hydrogel can be used to decrease the protein absorption and the growth of bacteria	[174]
	PHEA	Drug delivery; The silica particles were well dispersed in hydrogels; The release rate of aspirin decreased with the increasing content of silica	[175]
	PVA	Biomaterials; The tensile strength of PVA hydrogel increased with SiO_2_ NPs content. Proper content of SiO_2_ NPs could also enhance the permeability and swelling property, resulting in the improvement of the capillary water absorption capacity of PVA hydrogel	[176]
	CS	Bone tissue engineering; SiO_2_ NPs have a positive effect on cell viability and induce the occurrence of mineralization not only at the surface of the material but also in its entire volume; CS as bioactive injectable systems for bone tissue repair can undergo *in situ* gelation under physiological temperatures	[177,178]
	Peptide	Biosensor; PEG/peptide can form enzyme responsive hydrogel and collapse in response to diverse biological stimuli; The negatively charged silica NPs used are also at a sufficiently high concentration to form a regular periodic structure within the hydrogel, acting as a photonic bandgap which can reflect visible wavelengths of light	[138,140]
	Cellulose	Tissue engineering and cell cultures; SiO_2_ preserves extracellular matrix-like materials and cellular proteins; The intact 3D spheroids can be recovered from the hydrogel by a cellulase enzyme for downstream applications	[179]
	PMAA or PAA	SiO_2_ as adhesive fillers that interacted with PMAA chains can improved viscoelastic moduli (up to 8.7 times) and enhanced elasticity	[180,181]
	ALG or COL	Tissue engineering; SiO_2_ can improve the mechanical properties, including surface roughness and hardness, of the hydrogel	[182,183,184]
	PEG	Drug delivery; Hydrogel can form sustained-release depot systems; Incorporation of SiO_2_ NPs can regulate the pores size, gelation time and viscosity of the hydrogel; The drug loading capacity and drug release profiles could be tuned	[185,186]
MSNs	CS	Drug delivery; MSNs facilitate the drug loading and their subsequent release; CS enables pH-triggered drug release and improves the endocytosis of target cells and cell biocompatibility	[187]
		Drug delivery; The introduction of MSNs into CS will facilitate the gelation process at body temperature and also promote the elastic modulus; MSNs can load small drug molecules and then steadily release them for a long time period	[178]
	PClAETA	Biomaterials; Introduction of non-functionalized MSNs can improve the water absorption of the hydrogel	[188]
	Poly(aspartic acid)	Biomaterials; MSNs as crosslinker can optimize the pore morphology, improve thermal stability increase swelling ratio and enhance salt tolerance of the composite; MSNs can be uniformly and stably dispersed in the structure of the hydrogel	[189]
	PNIPAM	Biomaterials; MSNs can be used as effective “topological crosslinkers” to reinforce the PNIPAM hydrogels	[190]
HAP and nBG	GEL	Bone tissue regeneration; HAP significantly improves the stiffness of GEL hydrogels, while it maintains their structural integrity and swelling ratio; Introduction of HAP results in a lower swelling ratio, higher mechanical moduli, and better biocompatibility and promotes cell functional expression for osteon biofabrication; Gelatin hydrogels also provide natural cell binding motifs, making them amenable for 3D cell encapsulation; GEL provide cell-responsive characteristics, cell adhesion sites, and proteolytic degradability	[191,192,193]
	Silk fibroin	Bone tissue regeneration; HAP can increase compression modulus and mechanical properties, decrease the water uptake ability, improve metabolic and alkaline phosphatase activities of osteoblastic cells; Hydrogel plays regulatory role in oriented nucleation and growth of HAP crystals	[133,194,195,196,197]
	PAM	Tissue engineering; SiO_2_ can improve the mechanical properties, including surface roughness and hardness	[198]
	CS	Bone tissue regeneration; HAP enhances swelling, protein adsorption, exogenous biomineralization and osteoblast differentiation and also accelerates bone formation; Zn possessing excellent antimicrobial properties can tackle implant-associated microbial infections; CS can minimize immune response	[199,200,201,202]
	PVA	Bone tissue regeneration; Hydrogels are employed as a matrix gel in a mineralization solution; HAP was formed in the polymer matrix under mild conditions, mimicking mineralization in natural bone formation	[132,203,204]
	Pullulan	Bone tissue regeneration; Addition of HAP can improve compressive modulus of the scaffold, provide sites for cell adhesion, and render them osteoconductive *in vitro*; Hydrogels work as scaffolds for mineralization	[205]
	PECE	Tissue engineering; PECE endowed hydrogel good thermosensitivity and injectability; HAP can improve mechanical properties of hydrogel	[206]
	PEG	Bone substitute material; The formation of HAP in hydrogel matrices enable the acquisition of bioactive composites materials with desired shapes.	[207,208]
	ALG	Bone tissue engineering; Incorporation of nBG into hydrogel can combine excellent cellular adhesion, proliferation and differentiation properties, good biocompatibility and predictable degradation rates; ALG can increase lactate dehydrogenase and mitochondrial activity	[135,209,210,211]
	CHS	Bone tissue engineering; nBG shows an excellent stimulatory effect on bone formation; CHS improves integration of the nBG to prevent particle migration and promotes bone regeneration; The composite can encapsulate bone marrow to form a mechanically stable construct	[212]
	COL	Tissue engineering; Incorporation of nBG NPs improves mechanical stability and enhanced the proliferation rate and osteogenic differentiation	[136,213,214,215]
LAP	GEL/PAM	Tissue engineering; Incorporation of LAP can enhance thermal stability, tensile and stretching properties; GEL can significantly improve the hydrogel’s pH-responsive properties and enhance the antithrombogenicity but decrease the degree of hemolysis of the gels	[216]
	PEG/GEL	Tissue engineering; Hydrogel remain stable and provide a cell supportive microenvironment under normal cell culture conditions. LAP can preferentially induce osteogenic differentiation of human mesenchymal stem cells	[217]
	PEG	Tissue engineering; Incorporation of LAP significantly reduced the cure time while enhancing the adhesive and bulk mechanical properties of the hydrogel; PEG adsorbed onto LAP, forms a compact layer of mostly loops and trains on top of the nanoparticle and large loops around the edge of the particles; PEG elicits minimal inflammatory response and exhibits an enhanced level of cellular infiltration	[218,219]
	PAM	Tissue engineering; The incorporation of catechol on the PAM exhibits a strong affinity toward LAP and enhances stiffness and a viscous dissipation property	[220,221,222]
	P(MEOMA-OEGMA)	Drug delivery and tissue engineering; LAP as physical cross-linker had significant influence on the microstructure and swelling/deswelling behaviors of hydrogels.; OEGMA can increase equilibrium swelling ratio and water retention; Hydrogel can provide thermosensitivity and the excellent biocompatibility	[223]
	P(AM-DMAEMA)	Tissue engineering, cell culture substrates and biosensors; LAP serves as a physical cross-linker and can change the mechanical strength of the hydrogel under direct-current electric field	[224]
	PNIPAM	Tissue engineering; LAP can influence the polymeric chain arrangement and increase the mechanical toughness and thermal stability	[225,226]
	Pluronic F127	Tissue engineering; The interactions between LAP and Pluronic F127 may contribute to rearrangements of network structures at high deformations, leading to high elongations and improved toughness	[227]
	GMA	Biomaterials; LAP can reinforce mechanical toughness and elasticity; GMA regulates equilibrium swelling ratio and improves water content; Integrating LAP and GMA can improve self-standing ability and rheological, compression, and tensile properties	[228]
MMT	CS	Drug delivery; MMT can enhance the loading of positively charged drug and affect the hydrogel’s drug release mechanism and swelling properties; The hydrogel can be used for controlled- release of drugs	[137,229]
	CS-g-PAM	Superabsorbent polymer composites; Incorporation of MMT can optimize their absorption capacity, improve their swelling rate and salt-resistant ability; Composites exhibit moderate antibacterial activity in acidic medium	[230]
	PAM	Drug delivery; MMT effects the equilibrium swelling and drug release behavior of the composite; MMT can improve the barrier property of nanocomposite hydrogels and decrease the burse effect. MA effects the pH-responsivity on equilibrium swelling and release of drug	[231,232,233]
	P(ATC-AM)	Drug delivery; MMT serves as chemical cross-linker to enhance strength and toughness and decrease the swelling degree; ATC is cationic monomer and exist cation- exchange reaction with MMT	[234]
	GEL or COL	Drug delivery and wound dressing; Drug intercalation results in changes in MMT layered space; Integration of drug loaded MMT and gelatin creates biodegradable composite hydrogels with controlled drug release property, improved mechanical and thermal properties	[235,236]
	PAA or PMA	Biomaterial implants; MMT is used for adsorbing drug, achieving high drug loading; PAA is used for pH/bacteria- responsive releasing	[237]
	Polysaccharide	MMT can enhance strength and toughness and decrease the swelling degree	[238]
	PNIPAM	Tissue engineering; MMT can influence the polymeric chain arrangement and the pore size and increase the complex viscosity and adhesion strength as well as thermal stability	[239,240,241,242,243]
Other silicate particles	P(AM-IA)	Drug delivery; Incorporation of hectorite offers the hydrogel with suitable water absorbency, shear-resistance, high gel strength and good thermal stability; IA provides pH-responsivity on drug release	[244,245,246]
	ALG	Drug delivery; Halloysite nanotubes improve complex surface topography and structural integrity and achieve a sustained release of the growth factor; The composites enhance repair and regeneration in damaged or diseased tissues	[139]
	PEG/P(AA-VP)	Drug delivery; Phyllosilicate enhances the water uptake with a desirable strength; Hydrogels are used for pH responsible and controlled release	[247]
	PCL-PEG-PCL	Bone regeneration; Mesoporous magnesium silicate can enhance the compressive strength, elastic modulus, and hydrophilicity of hydrogel, and promote cell attachment and proliferation and increase the degradability of the hydrogel	[248]
	PAM	Tissue engineering; Attapulgite nanorods grafted with vinyl groups serve as macro-crosslinkers, which can significantly increase the modulus, strength, and toughness of hydrogels;	[249]
	P(AA-NIPAM)	Tissue engineering; The presence of imogolite nanotubes produced strong hydrogels that exhibit thermo-responsive volume transition because of the coil/globule transition of PNIPAM chains.	[250]

Mesoporous silica nanoparticles (MSNs); Hydroxyapatite (HAP); Poly[(2-acryloyloxyethyl) trimethylammonium chloride] (PClAETA); Zinc-doped chitosan (Zn-CS); nano-scaled bioactive Glass (nBG); Chondroitin sulfate (CHS); Laponite (LAP); 2-(2-methoxyethoxy) ethyl methacrylate (MEOMA); oligo(ethylene glycol) methacrylate (OEGMA); Guanidinium-pendant methacrylamide (GMA); Montmorillonite (MMT); Poly(acrylamide-co-maleic acid) (P(AM-MA)); (3-acrylamidopropyl) trimethylammonium chloride (ATC); Poly(ε-caprolactone)-poly(ethylene glycol)–poly(ε-caprolactone) copolymer (PCEC); Poly(acrylamide-co-itaconic acid) (P(AM-IA)); Poly(acrylic acid-co-vinyl pyrrolidone) (P(AA-co-VP)); Poly(ε-caprolactone)-poly(ethylene glycol)-poly(ε-caprolactone) (PCL-PEG-PCL).

### 3.4. Nanocomposite Hydrogels from Hydrogels and Metal NPs or Metal Oxide NPs

Hydrogels and metal or metal oxide NPs are two completely distinguished kinds of materials. Hydrogels are commonly made of natural and synthetic polymers, which possess numerous advantages over inorganic and metal materials, such as lower material cost and easier fabrication, better biocompatibility and biodegradability, and more versatile processability and functionalization. However, polymers usually display poor mechanical and thermal stability, and especially they lack some special functions, such as thermal, magnetic, optical and electrical conductivity [3]. With the increase of popularity of hydrogels in various biomedical applications including biosensing, bioimaging and biotracking/labeling, there is a growing need to engineer hydrogels with magnetic, electrical and optical properties. Metal and metal oxide NPs possess unique characters that are not commonly found in polymer materials, which have great potential as reinforcing elements to prepare composite hydrogels with unique characteristics and tunable properties [3,251]. Various types of metal and metal oxide NPs have been integrated into polymer hydrogels through a covalent or non-covalent fashion to fabricate nanocomposite hydrogels. Generally speaking, the incorporation of such NPs into hydrogel matrix by a covalent manner will lead to a relatively strong polymer-NPs interaction and thus change markedly the mechanical properties and swelling behaviors of hydrogels. On the other hand, there will be little impact on the viscoelastic and mechanical properties of the resultant hybrid hydrogels if the metallic NPs were incorporated into hydrogel networks by the weak non-covalent manner [251]. In this case, only the properties of the NPs, such as improved magnetic, optical, electrical conductivity and enhanced antimicrobial properties are added into the hydrogels. For example, the physical entrapment of Au NPs within the HA polymeric network did not improve the mechanical properties of hydrogel, due to the presence of only the weak interactions between polymers and NPs [252]. Nevertheless, significant increases in the stiffness were observed when surface-functionalized Au NPs can form covalent bonds to the polymer chains and crosslink with the polymeric networks [79,252,253].

A variety of metallic NPs, such as gold (Au), silver (Ag), platinum (Pt), cobalt (Co), nickel (Ni) and copper (Cu), and a series of metal oxide NPs including iron oxide (Fe_3_O_4_, Fe_2_O_3_), titania (TiO_2_), zinc oxide (ZnO), cupric oxide (CuO) as well as metal alloys and salts with desirable physicochemical properties were combined with natural and synthetic polymer networks and then were designed for diverse biomedical and pharmaceutical applications, including biological sensing, *in vivo* bioimaging, cell separation, drug delivery, conductive scaffolds, switchable electronics, and disease treatment [3,251]. We summarized the biomedical and biological applications of nanocomposite hydrogels from hydrogels and metal or metal oxide NPs, as shown in Table 3. Due to their unique functions and relatively simple preparation process, Ag, Au and magnetic iron oxide Fe_3_O_4_ NPs are the most widely investigated class of NPs for the fabrication of functional nanocomposite hydrogels.

Recently, Ag NPs with unique optical, electronic, and antibacterial properties have attracted much attention and have found many applications in field of medicine and pharmaceutics. One of the most attractive features of Ag NPs is their remarkably strong broad-spectrum antimicrobial activity in combination with a fairly low toxicity against human tissue [46,254,255,256,257,258]. However, because of strong dipole-dipole attractions between particles and high surface energy, the lack of sufficient physicochemical and dispersion stability in aqueous media limited their applications. As described above, hydrogels with large free space among the crosslinked networks can not only act as a reservoir for massive NPs loading, but also serve as a nanoreactor template for the nucleation and growth of NPs [259]. Embedding Ag NPs in crosslinked polymer networks to obtain Ag nanocomposite hydrogels has proved to be a most promising solution for stabilization and distribution problem, due to the excellent capacity of stabilizing NPs. Normally, Ag NPs can be introduced into the matrix of polymer hydrogel by simply blending the NPs with the preformed hydrogel, entrapping NPs during the swelling process or adding the NPs during the gelation process [260]. And Ag NPs can also be generated *in situ* in the swollen hydrogel, which can achieve a more uniform distribution of Ag-NPs [261,262]. As described in Table 3, the incorporation of Ag NPs into hydrogels will render them with high antibacterial activity and usually change their mechanical toughness, swelling ratio and stimuli responsiveness. Hydrogels work as an efficient stabilizer of Ag NPs and control the release of the Ag NPs. More importantly, because the biocompatible hydrogels can maintain a moist environment at the wound interface, act as a barrier to microorganisms, allow sufficient air and water vapor to permeate through, and absorb or remove excess exudates, as well as attach at the target site and be easily removed without trauma and pain, Ag NPs-hydrogel composites have great potential to provide functional coatings for various applications, such as wound dressings, catheters and bone cement [263,264,265]. It was worth pointing out that the inclusion NPs into natural polymer hydrogels, such as CS, GEL, COL and peptides can produce biodegradable composite materials, which can be employed as implantable dressings without the need of removal [266,267,268]. In addition, Abdel-Halim *et al.* [269] reported an electrically conducting nanocomposite hydrogel consisted of silver NPs and guar gum/poly(acrylic acid)hydrogel. The nanocomposite hydrogel’s electrical conductivity was found to be affected by the content of Ag NPs and the swelling ratio of the hydrogel. Endo *et al*. [256] developed a surface plasmon resonance (LSPR)-based optical glucose concentration sensor by incorporation of Ag NPs into pH-responsive hydrogels. The interparticle distances of the silver NPs in the hydrogel would be increased if glucose was present on the surface of this biosensor. And thus the absorbance strength of the LSPR was decreased, which can be used to specifically determine glucose concentrations.

**Figure 9:**
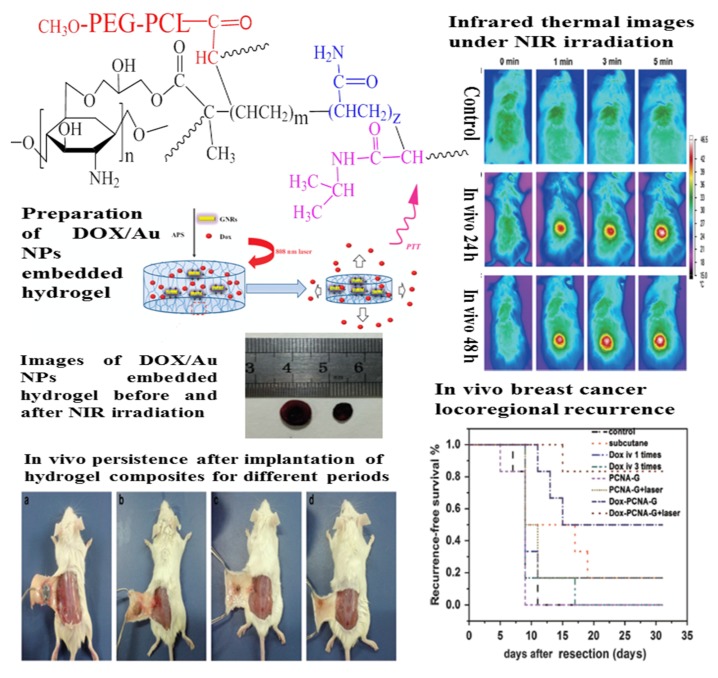
Schematic illustration of Doxorubicin (DOX)/Au NPs embedded hybrid hydrogels with the ability of photothermal therapy (PTT) and near infrared (NIR)-triggered thermo-responsive drug release, reproduced with permission from [270]. Copyright Nature Publishing Group, 2015.

Similarly, the inclusion of Au NPs within the hydrogel matrix has also emerged as a promising way to form a novel class of soft nanocomposites for various applications in the biomedical fields [270,271,272,273]. Au NPs with excellent optical and LSPR properties from the visible regime to the near infrared (NIR) have received intensive attention in biomedical applications. NIR light is very useful for biomedical applications because these wavelengths can penetrate deeply into biological tissues with relatively little attenuation and minimal damage, which has been wide used as a stimulus for photothermal therapy (PTT), photodynamic therapy (PDT), bioimaging and remote-controlled smart drug delivery [31,274,275,276]. One of the most remarkable properties of Ag NPs is that they can effectively absorb and scatter light due to LSPR and then efficiently convert optical energy into thermal energy [43,44,45]. Due to their very high photothermal conversion efficiency, Au NPs-hydrogel composites in combination with NIR laser irradiation have aroused tremendous interest as therapeutic agents for cancer therapy, especially for the PTT and PDT. Compared to other cancer therapies, PTT and PDT are both excellent minimally invasive therapeutic treatments [270,271,273,275,276]. For example, Wang *et al*. [275] reported a composite hydrogel that contained Au NPs, spinach extract, and PEGDA via *in situ* photopolymerization. Spinach extract can act as a photoinitiator to initiate the formation of the PEGDA hydrogel and serve as an excellent photosensitizer to generate cytotoxic singlet oxygen (^1^O_2_) with oxygen to kill tumor cells. Au NPs as a photoabsorbing agent can generate heat from optical energy to induce tumor tissue hyperthermia and accelerate the rate of ^1^O_2_ generation. The composite hydrogel shell on tumor cells can prevent the photosensitizer from migrating to normal tissue and keeps a high concentration on lesions, thereby enhancing the curative effect. More interestingly, a remote-controlled smart drug delivery can be easily achieved by NIR irradiating Au NPs-integrated temperature-responsive gel matrix. The gel structure will quickly collapse if the temperature rises above the lower critical solution temperature of the gel matrix due to the NIR-triggered photothermal effect. The fast response leads to an on demand burst release of drugs, which should be contributed to heat generation within the gel matrix, rather than external to the matrix [270,273,277]. Sershen *et al*. [277] firstly developed a photothermally modulated hydrogel using photoactive Au NPs in combination with thermal-responsive PNIPAM-PAM copolymers, which exhibited a controlled pulsatile release of methylene blue and proteins [277]. As shown in Figure 9, a novel chemo-photothermal co-therapy system was developed to prevent local breast cancer recurrence [270]. The temperature of the hybrid hydrogel system will be increased due to the photothermal effect of Au NPs under NIR irradiation, thus leading to a fast shrinkage of hydrogel and a significant increase in the release rate of loaded drug. This functional hydrogel effectively avoided breast cancer recurrence after primary tumor resection in a mouse model, due to the controlled administration of combination chemo-photothermal therapy and the decreased systemic toxicity. Besides using Au NPs based nanocomposite hydrogels for drug delivery and cancer therapy, they are also explored for biosensing [278], cellular therapy such as cell labeling and cell-biology research [279], and tissue regeneration [280].

**Figure 10:**
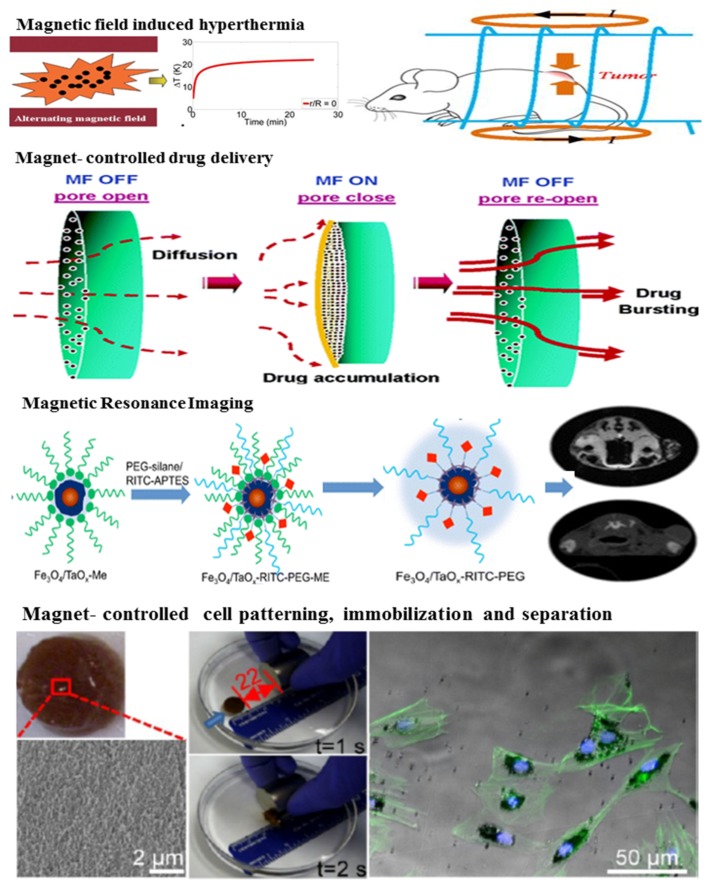
Schematic illustrations of typical biomedical applications of iron oxide NPs-hydrogel composites. Magnetic field induced hyperthermia, reproduced with permission from [286] Copyright Ivyspring International Publisher, 2012; Magnet-controlled drug delivery, reproduced with permission from [283]. Copyright American Chemical Society, 2006; Magnetic resonance imaging, reproduced with permission from [287]. Copyright American Chemical Society, 2012; Magnet-controlled cell patterning, immobilization and separation, reproduced with permission from [288]. Copyright American Chemical Society, 2015.

Unlike those noble metals NPs, some transitional metal, such as ferreous (Fe), cobalt (Co) and nickel (Ni) based NPs, exhibit excellent magnetic properties. Among them, pure iron oxides, such as maghemite (ɣ-Fe_2_O_3_) and magnetite (Fe_3_O_4_) NPs are most common magnetic nanomaterials for biomedical applications, due to their superior biocompatibility [47,48]. Moreover, the magnetic properties of these NPs can be tuned from ferromagnetic to superparamagnetic by adjusting their sizes, because of quantum size effects [47,48]. As shown in Table 3, the incorporations of these magnetic NPs into polymer networks also offer a high potential for several biomedical applications, such as magnetic field induced hyperthermia, magnetic resonance imaging (MRI), drug delivery, cell tracking and protein separation [47,48,281]. The typical biomedical applications of these magnetic hydrogel were shown in Figure 10. For example, the superparamagnetic property of iron oxide NPs can cause a temperature rise under alternating magnetic fields, and thus the use of magnetic induction hyperthermia for cancer therapy and cell-biology research has recently gained great interest. Samal *et al.* [282] introduced magnetic NPs into silk fibroin protein hydrogel scaffolds via dip-coating methods. The obtained magnetic scaffolds showed excellent hyperthermia properties achieving temperature increases up to 8 °C in about 100 s, improving the cell adhesion and proliferation. Magnetic induction hyperthermia also allows the on-off response of the system depending on temperature. Liu *et al.* [283] incorporated magnetic NPs into PVA matrix to create composite hydrogels, which have proven externally controlled on–off drug release from the hydrogels. The drug release behaviors can be remote-controlled by the use of a magnetic field, and the release profiles of drug were highly dependent on the particle size, strength of the magnetic field and the duration of the switching time. Composite hydrogels of thermo-sensitive PNIPAM with superparamagnetic NPs exhibited remotely-controlled thermal-responsive swelling behaviors in an alternating magnetic field [284]. The covalent conjugation of magnetic Fe_3_O_4_ NPs and bovine serum albumin (BSA) on the hydrogels allows these materials to be applied in magnetically assisted bioseparation processes [285].

Several other metallic NPs, metal-oxide NPs and metal alloys NPs can also be used to integrate with polymer networks to enhance the bioactivity of hydrogels. For instance, magnetic Co or Ni NPs have also been introduced into hydrogels to form ultra-flexible and strong magnetic actuator [289,290]. Some titania NPs and alumina NPs were incorporated within polymeric matrix to enhanced cell adhesion and proliferation for tissue engineering applications [291,292]. However, very limited studies focused on the use of these NPs to fabricate hybrid hydrogels for biomedical applications because of their potential toxicity and rapid oxidation. Overall, various types metallic and metal-oxide colloidal particles were incorporated with polymer network structures, which can impart them with exclusive optical, electrical or magnetic properties. The resultant nanocomposite hydrogels are highly suitable for different biomedical applications, especially for diagnostic and therapeutic applications.

**Table 3:** Summary of nanocomposite hydrogels from hydrogels and metal or metal oxide NPs for biomedical applications.

Fillers	Hydrogels	Applications and Functions	Ref.
Ag NPs	PAM	Wound dressing; Ag NPs demonstrate excellent antibacterial activity; Polymer hydrogels were used to keep the NPs stable and contact with skin	[254,255,293,294,295,296,297]
		Biosensor; Ag NPs can change the strength of the localized surface plasm on resonance by regulating interparticle distances; PAM can be swelling with ionic strength change	[256]
	PVP or PVA	Wound dressing or tissue engineering; Ag NPs are antibacterial agents and enhance the mechanical and thermal strength of the hydrogel; Hydrogels work as an efficient stabilizer of Ag NPs and control the release of the Ag NPs	[257,258,298,299,300,301,302]
	PNIPAM	Antibiotic materials; Ag NPs work as antimicrobial agents; PNIPAM is temperature-responsive component and make Ag NPs stable and uniformly distributed	[259,303,304,305,306]
	PEGDMA	Contact lenses; Ag NPs work as antimicrobial agents	[307]
	Acrylate-based copolymers	Biosensors and drug delivery; Ag NPs work as antimicrobial agents; Copolymer hydrogel with different swelling properties is used to disperse Ag NPs	[262,266,308,309,310]
		Ophthalmic lenses; The addition of Ag NPs is associated with the reduction of UV-B transmittance and increase in tensile strength	[311]
	AMPS-Na	Burn wound dressing; Silver hydrogel is efficient at preventing bacterial colonization of wounds and has good inhibitory action against bacteria	[263,312]
	NaDOC	Antibacterial materials; Ag NPs are antibacterial agents; NaDOC can immobilize Ag NPs via pH-induced self-assembly	[313]
	Graphene	Wound dressing; Ag NPs are antibacterial agents; Graphene is conductive to cellular adhesion and growth; The composite can significantly accelerate the healing rate of artificial wounds	[264]
	Silicone	Adhesion to catheters to prevent urinary tract infections; Ag NPs work as antimicrobial agents	[265]
	Lysine gelator	Nanomedicine; Gelator exhibits good gelation ability, low haemolytic activity and high biocompatibility to mammalian cells; Incorporation of Ag NPs exhibits excellent antibacterial activity and significant mechanical strength	[314]
	Peptides	Wound healing and sustained Ag NPs release; Ag NPs inhibit bacterial growth; Peptide fibers prevent aggregation of Ag NPs and improve the biocompatibility of the system	[267]
		Antibacterial materials and bioimaging; Fluorescent Ag NPs show excellent optical properties and antibacterial activity; Peptides make the Ag NPs stable	[315]
	PAA	Drug delivery; PAA is used to control the release of antibacterial drugs; Ag NPs can enhance the antibacterial activity	[316]
	Cellulose	Wound dressing and antimicrobial materials; Ag NPs show high antibacterial activity against Gram positive and Gram negative bacteria; Cellulose serves as a gelling agent as well as a reducing agent for AgNO_3_ and a stabilizer for Ag NPs	[247,261,317]
	GEL or COL	Wound dressing; Ag NPs are antibacterial agents; Hydrogel is used for stabling Ag NPs	[268,318,319]
	GEL/CS	Drug delivery and tissue engineering; Benzotriazole maleimide functionalized Ag NPs work as cross-linkers, improve the storage modulus and decrease swelling ratios	[320]
	AG-CS	Biosensor; Ag NPs improve the conductivity and mechanical strength of the hydrogels and also have antimicrobial activity; Biopolymer moieties act as both reducing and stabilizing agents	[321]
	AG	Antibiotic materials; Hydrogels promote the Ag NPs protection and inhibit their aggregation; Ag NPs can produce Ag + to kill bacteria	[322,323]
Au NPs	PNIPAM	Drug delivery; Au NPs can be used as a photoabsorbing agent to generate heat from optical energy; PNIPAM is thermo-responsive to control the release of drugs	[270,324,325,326]
		Biosensor for the detection of DNA; Au NPs immobilize DNA strands	[278]
	PEG	Tissue engineering; RGD modified gold nanoarrays as scaffold to control cell localization and differentiation	[279]
		Drug delivery; Au NPs are utilized as building blocks; α-CD and PEG form temperature-responsive reversible supramolecular hydrogel through host–guest interaction	[271]
	PEGDA	Tumor therapy; Au NRs can be used as a photoabsorbing agent to generate heat from optical energy; The introduction of Au NRs is conducive to the formation of the hydrogel and accelerates the rate of cytotoxic singlet oxygen generation; PEGDA can stabilize Au NRs	[275]
	PPy	Clinical diagnosis; Au NPs can further increase the specific surface area to capture a large amount of antibodies as well as improve the capability of electron transfer	[327]
	PEDOT	Electroporation-assisted cell uptakes; Au NPs work as microelectrode; PEDOT modification is effective in preventing electrolysis during the electroporation	[328]
	CS	Drug delivery; Au NPs act as a role of physical cross-linking agents; This physical cross-linked hydrogel shows excellent drug loading ability	[272]
	GEL	Bone tissue engineering; The hydrogels loaded with Au NPs promote proliferation, differentiation, and alkaline phosphate activities of human adipose-derived stem cells and have a significant influence on new bone formation; GEL is a photo-curable hydrogel to embed Au NPs	[280]
	Peptide	Tissue engineering; Au NPs act as cross-linking agents and strengthen the prepared hydrogel	[329]
	DNA	Drug delivery and photothermal therapy; Au NPs can generate heat upon laser excitation and be used for photothermal therapy; DNA hydrogel can be fragmented by heat generations to release drugs and Au NPs	[273]
	DNAzyme	Detection of lead; Au NPs are used for visual detection; DNAzyme can be activated by lead and conducive to dissolve the hydrogel to release Au NPs	[330]
	Graphene	Biosensor for nitric oxide detection; Au NPs catalyze the electrochemical oxidation of NO; Graphene is used for uniformly depositing Au NPs	[331]
Fe_3_O_4_ NPs	κ-carrageenan	Drug delivery; Fe_3_O_4_ NPs offer the hydrogel with magnetic-responsiveness properties; κ-carrageenan is pH responsive	[332,333,334,335]
	Starch	Drug delivery; Fe_3_O_4_ NPs endow the hydrogel with magnetic sensitivity; The composite also shows temperature/pH sensitivity	[336,337,338]
	Cellulose	Drug delivery; Magnetic nanoparticles are used as cross-linkers; The release of DOX is significantly enhanced at external magnetic field	[339,340]
	Fibrin	Tissue engineering; Fe_3_O_4_ NPs make the hydrogel show excellent hyperthermia properties under exposure to an alternating magnetic field; The presence of Fe_3_O_4_ NPs in the fibrin can improve cell adhesion and colonization of osteogenic cells	[282,341]
	HA	Magnetic resonance imaging and drug delivery; HA can be degraded by hyaluronidase and release hydrophobic drugs; Fe_3_O_4_ NPs serve as imaging agents to track the hydrogel degradation and degradation products *in vivo*	[342]
	CS	Drug delivery; The presence of Fe_3_O_4_ NPs can make the hydrogels have magnetic response property and deliver and release encapsulated anticancer agent at the tumor by the weak magnetic field	[343,344,345]
	ALG	Cell encapsulation; Alginate cell capsules with Fe_3_O_4_ NPs can be easily controlled and manipulated by external magnetic fields	[346]
	COL-HA-PEG	Cartilage tissue engineering; The presence of Fe_3_O_4_ NPs can make the hydrogel travel to the tissue defect sites under remote magnetic guidance	[288]
	Guar gum	Drug delivery and theranostic; The NPs work as imaging agents and produce hyperthermia; Aminated guar gum can form the hydrogel without using toxic crosslinking agents	[347]
	PPZ	Drug delivery and biodetection; Ferrite nanoparticles is used for long-term magnetic response theragnosis; The composite can be used for sustainedly releasing cargos	[348]
	PAM	Drug delivery and protein separation; The presence of Fe_3_O_4_ NPs can make the hydrogel exhibit higher dielectric constants and magnetic responsiveness; The composite also has the properties of fast adsorption	[349,350,351]
		Drug delivery; Incorporation of Fe_3_O_4_ NPs endows the hydrogel with the ability of direction-dependent thermogenesis in an alternating magnetic field; The novel magnetic hydrogel shows a direction-dependent release of drugs that has a 3.4-fold difference between the two directions	[352]
	PNIPAM	Drug delivery; Incorporation of magnetic nanoparticles endows the hydrogel with on-demand pulsatile drug release triggered by alternating magnetic field	[353]
	PVP or PVA	Drug delivery; Fe_3_O_4_ NPs are used for magnetic drug targeting; The hydrogel is used for carry cancer therapeutic agent	[354,355,356]
	PEGDA	Cell patterning; Incorporation of Fe_3_O_4_ NPs can manipulate space pattern of the hydrogel; PEGDA blocks are used as a stencil to define the area for cell adhesion and the second types of cells could be seeded after the magnetic block was removed to create heterotypic cell patterns	[357]
	Acrylate/acrylamide-based copolymers	Drug delivery and protein immobilization; Fe_3_O_4_ NPs endow the hydrogel with properties of magnetic stimuli-responsive; The copolymer hydrogel exhibits pH- and thermosensitive properties	[88,358,359,360,361]
	P(HEMA-GMA)-PANI	Immobilization of glucoamylase; Fe_3_O_4_ NPs show super-paramagnetism and make the hydrogel easily dispersed or separated from the medium without needing high magnetic intensity; Grafting of polyaniline on the hydrogel increases 3.4 times maximum adsorption capacity	[362]
Cu or CuO NPs	PVP/PVA	Bioimaging and drug delivery; Cu NPs with red emission properties are used for optical imaging as well as for flow cytometric probe of cellular uptake; Cu NPs also generate reactive oxygen species to enhance the efficiency of killing cancer cells; PVP is used for stabling the NPs and delivering drugs	[363]
	PEGDA	Antibacterial material; Cu NPs-modified hydrogel can be used for preventing bacterial infections	[364]
	Cellulose	Antibacterial material; CuO NPs endow the hydrogel with antibacterial activity	[365]
ZnO NPs	PAM	Wound dressing; ZnO NPs endow the hydrogel with antibacterial activity; PAM provides uniform distribution and binding of the NPs to the fiber surface and to prevent their agglomeration	[366]
	Cellulose	Antibacterial materials; ZnO NPs endow the hydrogel with antibacterial activity; The composite shows a pH and salt sensitive swelling behavior	[367,368]
	Chitin	Wound dressing; ZnO NPs endow the hydrogel with antibacterial activity and blood clotting ability; Incorporation of ZnO NPs improve healing rate and collagen deposition ability of composite bandages	[369]
ZnS NPs	PHEMA/PAA	Artificial cornea implants; ZnS NPs can improve the refractive index of the polymer	[370]
Pt NPs	Polyimide	Biosensor; Pt NPs work as microelectrades; The composite works as microsensor to detect glutamate *in vivo*	[371]
Ni NPs	Cellulose	Responsive biomaterials; Ni NPs offer the gel with magnetic response properties with can be changed by temperature and the size of the NPs	[289]
Co NPs	PHEMA	Tissue engineering; Co NPs enable the hydrogel that combine the use of saturation magnetizations and high particle loading, flexibility, and shape memory; PHEMA can reduce magnetic particle migration or loss	[290]
MnO_2_ NPs	CS	Biosensor; MnO_2_ NPs work as catalytic for H_2_O_2_; The composite can be used for detections of choline chloride	[372]
TiO_2_ NPs	PAM/PNIPAM	Responsive biomaterials; Modified TiO_2_ NPs serve as cross-linker and enhance the mechanical strength; The hydrogel bilayer is able to bend to opposite directions to form hydrogel circles in pure water and 20% NaCl solution	[373]

Poly(ethylene glycol diacrylate) (PEGDA); 2-acrylamido-2-methylpropane sulfonic acid sodium salt (AMPS-Na); Sodium deoxycholate (NaDOC); Agarose (AG); Polypyrrole (PPy); Poly(3,4-ethylenedioxythiophene) (PEDOT); Poly(organophosphazene) (PPZ); Polyaniline-grafted poly(2-hydroxyethylmethacrylate-co-glycidylmethacrylate) (P(HEMA-GMA)-PANI).

### 3.5. Nanocomposite Hydrogels from Hydrogels and Polymeric NPs or Liposomes

In comparison to conventional inorganic and low molecular weight organic materials, polymers exhibit unique properties, such as non-toxic and biocompatible nature, good physicochemical stability, excellent elasticity and ductility. Moreover, polymer chains are dynamic when polymers are conducted under usual conditions and particularly induced by an external environment, e.g., annealing, swelling and stretching, allowing for direct the self-assembly of NPs by use of polymers [32,34,374]. Especially because of the flexibility offered by macromolecular synthesis methods, the ability to respond to specific physiological or external stimuli, the almost infinite diversity of polymers in terms of property, architecture and composition, as well as their ease of functionalization, polymers have always been the first choice for the design and development of nanomaterials with tailored physicochemical and biological properties [32,34,374]. A wide variety of polymeric NPs, such as polymeric micelles, nanogels and particles, dendrimers as well as liposomes, have been developed and gained great attention in biomedical and pharmaceutical applications. The inclusion of these polymeric particles into a hydrogel system has been explored to improve the performance of hydrogels and polymer NPs [3]. This strategy strongly has provided the opportunity to obtain nanocomposite hydrogels with undoubtedly improved properties and performances. The advances in the fabrication of polymer NPs and hydrogels composites and their applications in drug delivery, tissue engineering, and other biorelated domains were summarized in Table 4. In addition, some important studies in this topic were highlighted in detail and shown as below.

**Table 4:** Summary of nanocomposite hydrogels from hydrogels and polymeric NPs or liposomes for biomedical applications.

Fillers	Hydrogels	Applications and Functions	Ref.
PLGA particles or NPs	PVA	Loading and delivery of biomacromolecular drugs; PLGA can increase drug loading and decrease burst release; Hydrogel can prevent the foreign body reaction(FBR), promote angiogenesis around subcutaneous implants and extend the lifetime of implantable biosensors	[375,376]
		Drug delivery; PVA hydrogel acts as a hydrophilic base to support the microspheres; PLGA microspheres serve as drug reservoirs to continuously drug release	[377,378,379,380,381]
	ALG	Drug delivery; Maintain consistent release of rhBMP-2; Improve bone formation and osseous integration	[382]
	PuraMatrixTM peptide	Drug delivery; PuraMatrixTM peptide hydrogel as vaccine adjuvants to recruit and activate immune cells	[383]
	F127	Drug delivery; Sustained release of protein drugs; Temper burst release and prolong delivery of drugs at the site of a spinal cord injury	[384,385]
	P407	Tissue engineering; Preserve protein structure and integrity, allowing a better and prolonged release profile and the maintenance of their biological activity. Hydrogel further protected it from the hydrophobic environment	[386]
	ALG	Cell-based tissue engineering; Offer a continuous and localized release of drug; Provide a physical support for microcapsules, facilitating administration, ensuring retention and recuperation and preventing dissemination; Reduce post-transplantation inflammation and foreign body reaction, thus prolonging the lifetime of the implant	[387]
	PEI-PEGDA	Drug delivery; Hydrogels are used for carrying dual or multi-molecular compounds and releasing them in a bimodal, sequential manner	[388]
	HAMC	Drug delivery; HAMC hydrogel is well-tolerated, having a minimal inflammatory response; NPs offer sustained release while the HAMC gel localizes the NPs at the site of injection	[389,390,391,392,393,394,395]
	PAM	Antivirulence treatment of local bacterial infection; RBC-coated NPs is an effective detoxification platform against bacterial infections; Hydrogels preserve the structural integrity and the functionalities of the contained NPs and offer additional engineering flexibility to improve the therapeutic efficacy	[396]
	MC	Drug delivery; Particle localization, decreases initial burst, and further prolongs release	[397]
	GG	Sustained local delivery of drug; For the local treatment of osteoporosis and other bone tissue disorder; Improve the low bioavailability and decrease the high toxicity of sodium alendronate	[398]
	PNIPAM	Drug delivery; PLGA used for the isolation of the drug, a slower drug-release rate, and the achievement of different drug release profiles	[399]
	PAMAM	Drug delivery; The residence time of pilocarpine can be prolonged by hydrogel; PLGA nanoparticles are safe for delivery of ophthalmic agents and are capable of sustained delivery of antiglaucoma agents	[400]
	Fmoc-peptide	Drug delivery and tissue engineering; Peptide hydrogel will likely be more stable *in vivo*; Sustained release of drug	[401]
DBM-BHPEA NPs	Silicone	Protect the eyes from UV rays; Increase the pore size, allowing the particles to diffuse into the lenses.	[402]
HPMC-PEG NPs	Branched PAA	Drug delivery; Optimize drug therapies in which the release of hydrophobic compounds; Selectively direct to specific cell lines; Able to stay localized at the injection site	[403]
Silk fibroin particles	Silk fibroin	Tissue engineering; Offering well suited rheological features for injectability, and shape conformability into defect sites as well as controlled delivery rate	[404]
PHBV NPs	GG	Drug delivery; Minimally invasive administration and the controlled delivery of the active agent	[405]
PEG-PLA NPs	HPMC	Drug delivery; Shear-thinning and self-healing properties; Dual loading of a hydrophobic molecule into the NPs and a second hydrophilic molecule into the aqueous bulk of the gel, and thus enabled simultaneous release of both hydrophobic and hydrophilic drug *in vivo* from a single gel.	[406]
PPy NPs	ALG	Drug delivery and tissue engineering; PPy is stable in solution over the period of a month, and have good drug loading capacity; Localized depot releasing systems; Pulsatile releasing behaviors and maintain morphology and mechanical strength.	[407,408]
PHBHHx NPs	CS/GP	Drug delivery; Relatively strict control of long-term insulin release	[409]
PGT NPs	Silicone	Drug delivery in eye; Increase patient compliance and the bioavailability; Increase the release duration from the contact lenses; Establish safety and efficacy of glaucoma therapy by extended wear of nanoparticle loaded contact lenses	[410]
CS NPs	GX	Scaffold system for dual growth factor delivery in bone regeneration; Develop an *in situ* gelling biomaterial combining the viscoelasticity of natural polymers with the powerful antimicrobial properties of chitosan	[411]
PMMA NPs	Polysaccharide-PAA	Drug delivery; Hydrogels can remain localized at the site of injection, showing high biocompatibility and good ability to provide short term delivery. PMMA based NPs can be traced both *in vitro* and *in vivo* biological studies over a long period of time without side effects due to the biodegradation process	[412]
PβAE NPs	PAMAM-DEX	siRNA delivery; High transfection efficiency and low cytotoxicity; Sustained delivery of the siRNA; Enhance the stability of the NPs	[413]
PLA particles	PECE	Drug delivery and tissue engineering; Increase the thickness of the corium; Increase cell adhesion of microspheres	[414]
PAMAM dendrimer	CS-PEG	Drug delivery; Dendrimer was used to increase the solubility, loading efficiency and homogeneity of hydrophobic drug; Hydrogel provide a local drug delivery depot for a prolonged drug release	[415]
	COL	Drug delivery; Dendrimer can improve the biostability and structural integrity of COL, which can make COL have higher denature temperature and resistance against collagenase digestion	[416]
	HA	Biofabrication; Form a fast cross-linking hydrogel; Improved the cell viability, proliferation, and attachment	[417]
	PEG-LA-DA	Tissue engineering; The multiple cross-linking sites present on the dendrimers can increase the cross-linking density at lower concentrations; The spherical dendrimers may provide discrete “molecular islands” in the network to limit swelling and improve mechanical properties; The multiple end-groups on the dendrimers facilitate the introduction of functional groups into the system at the nanoscale level	[418]
	Carbopol 980	Drug delivery; PAMAM dendrimers strongly affects their influence on the improvement of solubility and antifungal activity of drug	[419]
	PEG	Tissue engineering and drug delivery; Hydrogel crosslinked with dendrimers, showing improved cytocompatibility, controlled swelling and degradation	[420]
Solid lipid NPs	Poloxamer	Drug delivery; NPs can increase gel strength and mucoadhesive force; Easy to administer rectally; Gelled rapidly inside the body; Increased dissolution rate of the drug; Reduced initial burst effect	[421]
Liposomes	PAM	Drug delivery and antibacterial; Hydrogel can stabilize liposomes against fusion and preserves the structural integrity of the liposomes; The hydrogel formulation allows for controllable viscoeleasticity and tunable liposome release rate	[422]
	Peptide	Tissue regeneration; Liposomes can enhance binding of growth factors to peptide fibers of the gel matrix and achieve delayed release; Bimodal drug release	[423]
	HA	Drug delivery; Strengthen the network formed by HA chains, high efficiency encapsulation, easily injectable and less invasive, delay and control the release of drugs for local delivery	[424,425,426,427]
	PNIPAM	Drug delivery; The intact liposomes with their content can be controlled to release from hydrogel by changing the temperature, which can be used for temperature-triggered on-demand delivery	[428]
	PVA	Tissue engineering; Hydrogel can retain the structural integrity and contents of liposomes	[429]
	PVP-PAA-PBMA	Drug delivery, Hydrogel can ensure liposomes original vesicle structure; Liposomes can improve viscoelastic features and drug release profiles	[430]
Micelles	PECA	Drug delivery; Improve docetaxel solubility and permeability; The pH-responsive hydrogel controlled the micelles diffusion excellently in intestinal environment, thus achieving the target delivery of drug-loaded micelles to small intestine and significantly improvement of the oral bioavailability of docetaxel	[431]
	PEG-PCL-PEG	Drug delivery and wound dressing; High drug loading and encapsulation efficiency; Improve combined curcumin solubility and permeability; Exhibit excellent wound healing activity and promote tissue reconstruction processes	[432]
	Agarose	Drug delivery; Achieve functional hydrogels capable of stimulus-triggered drug release	[433]
	PEG	Gene delivery; The incorporation of the nanosized micelles provided an excellent mean to tune physical properties of the hydrogels, such as increasing porosity and tunable mechanical property of the hydrogels and providing the best balance among hydrogel stiffness and porosity for cell survival	[434]
Nanogels or microgels	PEG	Tissue engineering; Hydrogel can control the degradation and release of nanogels; Possess relatively strong mechanical properties, biodegradability; Nanogels can trap proteins by hydrophobic interactions; Sustainedly release proteins in their native form	[435]
	PAM	Obtain macroscopic hydrogels with a fast response to external stimuli; Obtain strong hydrogel matrixes and composite gels with an internal structure	[436]
	PEG	Drug delivery; Multidrug delivery system; High drug loading and encapsulation efficiency; Encapsulate various hydrophobic substances	[437]
	DEX	Drug delivery; Both proteins and poorly water-soluble low-molecular-weight drugs can be efficiently encapsulated in the nanogels; Eliminating the initial burst release; Offer a maximum pharmacological efficiency at a minimum drug dose, reducing administration frequency and improving patient compliance	[438]
	Carbohydrate	Drug delivery; Improve loading or prolong delivery of a target drug; Prevent migration of the microgels from target sites; Provide an additional diffusive barrier for drug release; Reducing burst release effects and prolonging drug release kinetics	[439]
CHP nanogel	Hyaluronan	Drug delivery; Minimize denaturation by trapping the protein in a hydrated polymer network; Simultaneously achieved both simple drug loading and controlled release with no denaturation of the protein drugs	[440]
Vesicles	CS	Functional biomaterials; The vesicle serves as reversible “dynamic” crosslinks that hydrophobic chains can be either embedded into the bilayers of vesicle or pulled out; Vesicles can afford multi-action sites for hydrophobic interaction, indicating that the self-healing rate of such a hydrogel would be much faster	[441]
	PVA	Drug delivery; The vesicles can be evenly dispersed in PVA and are stable. The release can be triggered and the calcein diffuses afterwards quickly throughout the gel	[442]
	Xanthan	Drug delivery; The hydrogel shows a protective effect on vesicle integrity and leads to a slow release of the loaded model molecules from the polysaccharidic system; The vesicular structures may enhance the delivery of drug in the stratum corneum due to their specific constituents	[443]
Micro-droplet	ALG	Drug delivery and bone tissue engineering; Facilitate the regional regulation of encapsulated cell fate; *In situ* formation of localized, sustained bioactive molecule delivery to encapsulate stem cells for therapeutic applications	[444]
Virus	ALG	Regenerative medicine; Significant improvement in cell attachment; Mimic the biological niche of a functional tissue; Localization, delivery, and differentiation of stem cells	[445]

Poly(D,L-lactide-co-glycolide) (PLGA); Cholesterol-bearing pullulan (CHP); Gellan gum (GG); Poly(ethylene glycol) (PEI); Poly(ethylene glycol) dimethacrylate (PEGDMA); Hyaluronan/methyl cellulose (HAMC); Methylcellulose (MC); Polyamidoamine (PAMAM); Hydroxypropylmethyl cellulose derivatives (HPMC); 1,3-diphenylpropane-1,3-dione (dibenzoyl methane) (DBM); 2-(4-Benzoyl-3-hydroxyphenoxy)ethyl acrylate (BHPEA); *N*-acetylglucosamine(GlcNAc); Poly(hydroxybutyrate-co-hydroxyvalerate) (PHBV); Poly(-lactic acid) (PLA); Polypyrrole (PPy); Poly(3-hydroxybutyrate-co-3-hydroxyhexanoate) (PHBHHx); β-glycerophosphate disodium salt (GP); Propoxylated glyceryl triacylate (PET); Gellan xanthan (GX); Poly(methyl methacrylate) (PMMA); Oligopeptide-terminated poly(beta-aminoester) (PβAE); Poly(ethylene glycol)-poly(e-caprolactone)-poly(ethylene glycol) (PECE); Hydroxyethyl cellulose (HEC); Poly(lactic acid)-b-poly(ethylene glycol)-b-poly(lactic acid) with acrylate end-groups (PEG-LA-DA); Dual-reverse thermosensitive hydrogel (DRTH); Poly(2-vinyl pyridine)-b-poly(acrylic acid)-b-poly(n-butyl methacrylate) (PVP-PAA-PBMA); PEG-(Poly(ε-caprolactone-co-trimethylene carbonate)) (PEG-P(CL-co-TMC)); Polyethylene glycol dimethacrylate (PEGDMA); Poly(ε-caprolactone)-poly(ethylene glycol)-poly(ε-caprolactone) copolymer (PCEC); Poly(ethylene glycol)–poly(ε-caprolactone)-acryloyl chloride (PECA); Poly(ethylene oxide) (PEO)-b-poly(2-(*N,N*-diisopropylamino)ethyl methacrylate) (PEO-PDPAEMA); Polycarbonate (PC); Acryloyl group modified cholesterol-bearing pullulan (CHPOA); Dextrin (DEX); Cholesterol-bearing pullulan (CHP).

**Figure 11:**
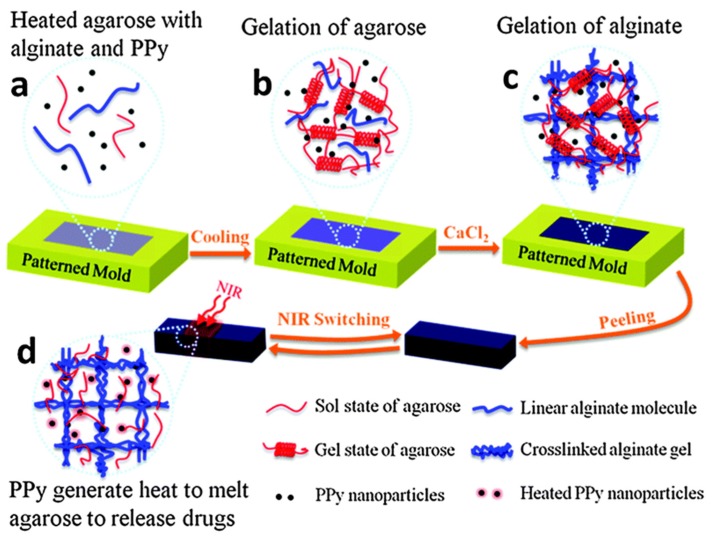
Schematic illustration of a NIR laser responsive deformation-free hydrogel containing embedded polypyrrole (PPy) NPs: (**a**) pre-heated agarose solution was mixed with alginate and PPy NPs and injected into patterned mold; (**b**) cooling to induce gelation of agarose, determining shape of the hydrogel and served as a template for alginate gelation; (**c**) CaCl_2_ was added to form an alginate network, which provided the mechanical support during laser irradiation; (**d**) periodic switching of NIR laser to digitally control agarose melting for pulsatile drug release, reproduced with permission from [408]. Copyright Royal Society of Chemistry, 2014.

Polymeric NPs have obtained great attention in drug delivery applications because of their capability to entrap hydrophobic or hydrophilic drugs. Among the various polymer materials used in drug delivery and biomedical devices, poly(lactic-co-glycolic acid) (PLGA) has attracted most considerable attention because of its attractive properties, such as excellent biocompatibility and biodegradability, European Medicine Agency approval and FDA in drug delivery systems. Typically, PLGA-based polymeric NPs have been integrated into different hydrogels and the resulting nanocomposite hydrogels have shown promise to develop novel biomedical materials with useful functions. Lampe *et al.* [446] entrapped PLGA microparticles within a PEG-based hydrogel to locally release two types of neurothropic factors with different release profiles. PLGA microparticles incorporated hydrogels possessing coordinated drug delivery ability can release two different neurothropic factors in different regions of the brain, significantly reducing the microglial response relative to sham surgeries, and thus providing a useful tool for further development in combining drug delivery and tissue engineering. Hydrogels have been extensively investigated as a carrier for loading and local delivery of various hydrophilic molecular cells and compounds. To tailor the release rates of multiple drugs and also manipulate the phenotypic activities of co-cultured cells in a hydrogel system, Yonet-Tanyeri *et al*. [388] separately incorporated two isoforms of vascular endothelial growth factor (VEGF121 and VEGF165), into the poly(ethylene imine)-poly(ethylene glycol) diacrylate (PEI-PEGDA) gel and PLGA microparticles. And then the PEI-PEGDA gel and PLGA microparticles were embedded in the poly(ethylene glycol) dimethacrylate PEGDMA hydrogel. The PLGA microparticles were used as the VEGF165 carrier to limit initial VEGF165 burst. PEI-PEGDA gel was used as VEGF121 carrier. Apparently, a bimodal, sequential macromolecular release profile could be attained, including rapid VEGF121 release with the degradation of the PEI-PEGDA gel and sustained VEGF165 release from the PLGA microparticles. A novel polymer coating consisting of PLGA microspheres dispersed in PVA hydrogels was developed in combination with dummy sensors as a “smart” drug eluting coating with high biocompatibility for implantable biosensors to avoid the foreign body response, and thus improve sensor performance *in vivo* [376]. The polymeric microspheres slowly release tissue-modifying drugs at the implantation sites to control the inflammation and fibrous encapsulation, while the hydrogel allows rapid analyte diffusion to the sensing elements. Samanta *et al.* [407] reported a generalized pH-sensitive drug delivery system based on polypyrrole nanoparticles (PPy NPs) with good drug loading capacity, which can be immobilized in a calcium alginate hydrogel, allowing for sustained release of the incorporated drug for more than 21 days. Luo *et al.* [408] synthesized a kind of NIR laser responsive material from agarose/alginate double network hydrogels composited with PPy NPs. As shown in Figure 11, the combination of alginate and agarose was synergistic. The agarose/PPy network provides the hydrogel with capability of responding to light. On the other hand, non-thermal-responsive alginate keeps the mechanical properties of the hydrogel during irradiation. Moreover the agarose hydrogel network was able to form a supporting template for Ca^2+^ diffusion control, which regulated alginate gelation processes and forms objects with shapes. As a result, the hybrid hydrogel exhibited pulsatile releasing behaviors according to the laser switching while maintaining its morphology and mechanical strength.

**Figure 12:**
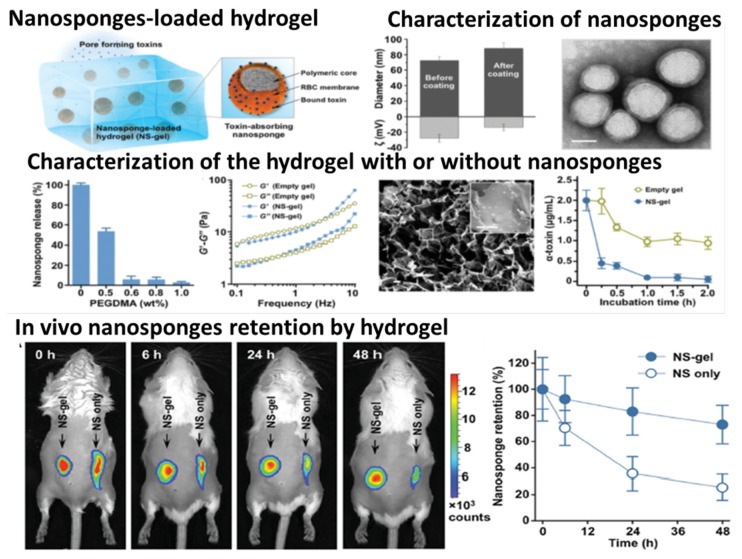
Schematic illustration of a hydrogel retaining toxin-absorbing nanosponges (NS-gel) for local treatment of bacterial infection. The toxin nanosponge was constructed with a poly(D,L-lactide-co-glycolide) (PLGA) core wrapped in natural red blood cell (RBC) bilayer membrane and was subsequently embedded into polyacrylamide (PAM) hydrogel, reproduced with permission from [396]. Copyright John Wiley and Sons, 2015.

Wang *et al*. [396] reported an advanced hybrid hydrogel that integrates toxin nanosponges (red blood cell (RBC) membrane-coated PLGA nanoparticle system) with PAM hydrogel for local treatment of bacterial infection, as shown in Figure 12. The hydrogel can effectively withhold the nanosponges in its matrix without compromising toxin transport for neutralization. After subcutaneous injection to mice, the hybrid hydrogel can also effectively anchor the nanosponges at the injection sites (a property facilitating toxin absorption), showing significant antivirulence therapeutic efficacy. This study demonstrated the incorporation of the nanosponges into hydrogel provide a new and effective detoxification strategy for the treatment of localized bacterial infection. In addition of PLGA particles, a wide variety of polymeric NPs or microparticles have been used to combine with hydrogel system to form nanocomposite hydrogels with unique physicochemical and biological properties, as shown in Table 4. These approaches have provided a promising novel platform for drug delivery and tissue engineering, especially for sustained delivery of multiple drugs at respective rate.

**Figure 13:**
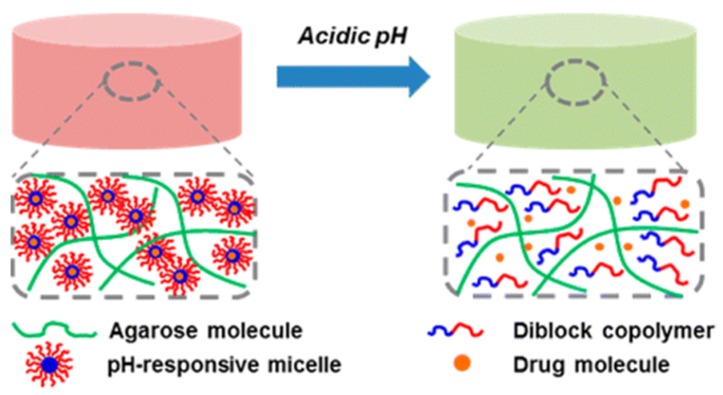
Schematic illustration of agarose hydrogels embedded with pH-responsive diblock copolymer micelles for triggered release of substances, reproduced with permission from [433]. Copyright American Chemical Society, 2013.

Polymer micelles are a typical type of nanoscale polymeric particles, which are generated from self-assembly of amphiphilic block-copolymers in water. They present a core-shell structure wherein the hydrophilic corona imparts colloidal stability against aggregation while the hydrophobic core can serve as a microenvironment for the incorporation of drugs. Due to their abundant chemical diversity, functionality and tunable physicochemical properties, micelles are one of the most versatile nanocarriers for the delivery of hydrophobic drugs [34]. The incorporation of the nanosized micelles into hydrogels provides not only a multiple drug delivery system, but also an excellent means to tune physical properties of the hydrogels. Jin *et al*. [433] developed pH-responsive block copolymer micelles as drug carriers for use in hydrogel scaffolds for neural repair, as shown in Figure 13. These micelles are formed from PEG-b-poly(2-(*N,N*-diisopropylamino)ethyl methacrylate) (PEG-b-PDPAEMA) with pH sensitivity due to the tertiary amine groups of PDPAEMA. The incorporation of these micelles into agarose hydrogels to obtain hybrid hydrogels with capable of stimulus-triggered drug release, which could allow the controlled delivery of hydrophobic neuroprotective agents to improve survival of transplanted cells in tune with signals from the surrounding pathological environment. Micelles have also been incorporated into hydrogels to enhance their mechanical properties. Xiao and coworkers [447] covalently incorporated polymer self-assembled micelles into PAM hydrogel. The mechanical properties of the micelle-linked PAM hydrogel can be regulated by adjusting the amount of micelles. 4-fold increase in Young’s modulus and 2-fold increase in tensile stress were observed when the concentration of micelles was increased from 7.5 to 15 mg/mL.

**Figure 14:**
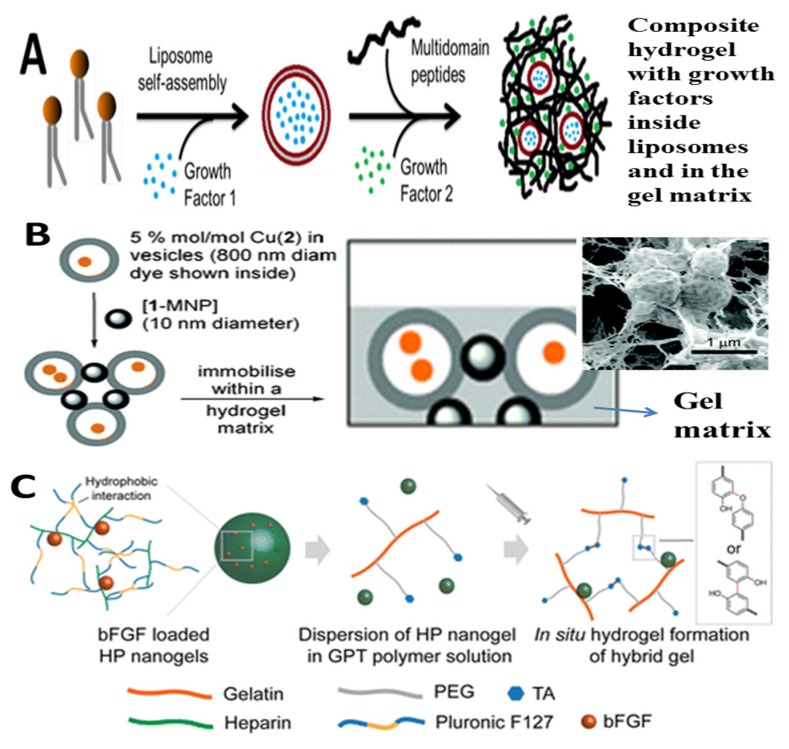
Schematic illustrations of nanocomposite hydrogels (**A**): from hydrogel and liposomes, and the formation of hybrid hydrogels by stepwise orthogonal self-assembly, reproduced with permission from [423]. Copyright American Chemical Society, 2014; (**B**): from hydrogel and vesicles, the formation of hybrid hydrogels by the sol–gel transition of vesicle-embedded composite hydrogels, reproduced with permission from [452]. Copyright Royal Society of Chemistry, 2009; (**C**): from hydrogel and polymer nanogels, and the formation of hybrid hydrogels via an enzymatic reaction, reproduced with permission from [453]. Copyright American Chemical Society, 2014

Liposomes are a kind of micelles from the self-assembly of phospholipids dispersed in an aqueous environment. These vesicles contain an aqueous volume entirely enclosed by a lipid bilayer membrane. Manipulation of composition, size, charge and lamellarity of liposomes allow the entrapment of payload both in the aqueous compartment and within the membrane. Hydrogels embedded with liposomes may generate a drug delivery system with desirable features and superior performance. Weiner *et al.* [448] may be the first to introduce liposomes into collagen gel. They observed that the release rate of payloads encapsulated in liposomes decreased in the presence of the hydrogel. The use of liposomes for drug incorporation within the matrix of hydrogel contact lenses is especially promising due to their thermodynamic stability and high drug-loading capacity [449]. Especially, this kind of nanocomposite hydrogels enables the possibility of achieving a controlled and sustained drug release in the eye without compromising the optical performance. In other words, this strategy can avoid the major limitation of common dosage (eye drops), which is the extremely short residence time of drug in the cornea because of the rapid clearance and the dilutive effect of tears [449]. Gulsen *et al.* [450] encapsulated various drugs in liposomes consisting of dimyristoyl phosphatidylcholine (DMPC) followed by incorporation into the hydrogel contact lenses. It was found that these hydrogel can control drug release for up to 8 days. Wickremasinghe *et al.* [423] created a unique nanocomposite hydrogel by stepwise self-assembly of liposomes and multidomain peptide fibers, as shown in Figure 14A. The two-component system can achieve controlled release of desired growth factors and cytokines over multiple time scale. This bimodal release system would have great potential in the field of tissue engineering and regenerative medicine.

Polymer vesicles, usually called polymersomes, are sphere-like shell structures in which an aqueous compartment is enclosed by a bilayer membrane generatedfrom the self-assembly of amphiphilic block copolymers [451]. In comparison with liposomes, polymer vesicles possessing better stability, higher toughness, tailorable membrane properties are attractive candidates for applications including drug delivery, encapsulation and nanoreactors. Polymeric vesicles have also been embedded in hydrogel to prepare functional nanocomposite hydrogels. For example, Litvinchuk *et al*. [442] reported a long-term stability of poly(2-methyl oxazoline-block-polydimethylsiloxane-block poly(2-methyl oxazoline) (PMOXA-PDMS-PMOXA) vesicles loaded in PVA hydrogels. The study found that the vesicles in the hydrogel are stable and the drugs could diffuse quickly throughout the PVA hydrogel, providing a promising controlled release system for water soluble drugs. Hao *et al.* [441] developed a thermal-responsive self-healing nanocomposite hydrogel by mixing hydrophobically modified chitosan with vesicle solution. This vesicle-based nanocomposite hydrogel exhibited an interesting self-healing feature in a matter of seconds through the autonomic reconstruction without the use of healing agent. Mart *et al.* [452] reported a new type of tissue-mimetic material, magnetically-sensitive vesicle-embedded composite ALG hydrogels. These vesicle gels contain vesicles crosslinked by Fe_3_O_4_ magnetic nanoparticles, as shown in Figure 14B. And these magnetic nanoparticle–vesicle assemblies render the prepared hydrogels the ability of hosting membrane-bound enzymes/glycolipids and responding to alternating magnetic fields to reach a magnetic trigger-controlled release of stored drugs or bio(macro)molecules, leading to potential applications as smart scaffolds for stem cells or remotely triggered *in vivo* drug delivery systems.

Typically, nanogel, a nanosized crosslinked polymer network, is an ideal nanocarrier for biomacromolecular drugs, as it can be effective in minimizing protein-denaturation by trapping the protein drug in a hydrated microenvironment. In one study, protein-loaded nanogels were physically encapsulated into a hyaluronan (HYA) hydrogel system for sustained delivery of protein [440]. The proteins were spontaneously trapped into the nanocomposite hydrogel without any obvious denaturation by the physical entrapment of nanogels in the HYA gel. The long-term release of protein both *in vitro* and *in vivo* was achieved by controlling the density of the HYA gel. This study offered a biocompatible, safe and sustained release formulation for proteins without the problem of denaturation of proteins in the formulation and during the formulation process. Park *et al.* [453] reported a hybrid hydrogel system composed of an *in situ* cross-linkable gelatin-poly(ethylene glycol)-tyramine (GPT) hydrogel and a self-assembled heparin-pluronic (HP) nanogel. As shown in Figure 14C, the hybrid hydrogels were prepared via enzymatic reaction in the presence of horseradish peroxidase and hydrogen peroxide. The injectable GPT hydrogels can play a role as a physical bulking reagent and the growth factor (GF)-loaded HP nanogels allow sustained release of GFs from the matrix to improve urethral tissue regeneration. The results indicated that the hybrid gel matrix can release GF continuously up to 28 days and stimulate the regeneration of the urethral muscle tissue around the urethral wall, which may offer an advanced therapeutic method for treating the urinary incontinence as well as an application for regenerative medicine.

Among the various types of polymeric NPs, dendrimer, a synthetic polymeric macromolecule of nanometer dimensions, are particularly fascinating, due to its unparalleled molecular uniformity, narrow molecular weight distribution, defined size and shape characteristics, highly branched structure, available internal cavity as well as a multifunctional terminal surface. These physicochemical characteristics of dendrimers, especially the multitude of functional groups on their periphery and abundant cavities within their interior, lead to higher reactivity and loading efficiency compared to the linear polymers, which make these macromolecules appropriate candidates for drug delivery [417,454,455]. Dendritic nanoparticles can also be incorporated into the hydrogel network via covalent or non-covalent interactions between dendrimers and the polymeric chains to obtain reinforced nanocomposite hydrogels with specific performance. For example, Desai *et al.* [420] developed a photocurable PEG hydrogel crosslinked with polyamidoamine (PAMAM) dendrimers, and the cytocompatibility, swelling and degradation of the resulting nanocomposite hydrogels can be controlled by modification of the chain length and charge density of PAMAM dendrimers. In another study, PAMAM NPs were physically integrated within COL scaffolds to improve the mechanical stiffness and structural integrity [416]. Interestingly, the human conjunctival fibroblast proliferation in COL hydrogels was significantly improved due to that the addition of PAMAM NPs can increase the stiffness of nanocomposite network. The use of these dendrimers-based nanocomposite hydrogels are envisioned in biomedical and pharmaceutical fields that require porous structure with controlled drug release properties [3]. Söntjens *et al.* [456] reported a novel hydrogel scaffold for cartilage repair, based on a water-soluble and multivalent triblock copolymer consisting of methacrylated poly(glycerol succinic acid) dendrimer terminal blocks and a poly(ethylene glycol) core. The terminal methacrylates allow biocompatible and mild photo-crosslinking with a visible light, which will facilitate *in vivo* filling of irregularly shaped defects with the dendrimer-based scaffold. The multivalent dendrimer components allow high crosslink densities, which will inhibit the swelling after crosslinking and simultaneously introduce biodegradation sites. The degradation properties, mechanical properties and water content of the hydrogel can easily be tailored by changing the biodendrimer concentration. Zhang *et al.* [457] reported a photocrosslinkable hyperbranched polyesters (HEP)-based hydrogel with sustained drug release profiles for cellular therapies. These photocrosslinkable HPE not only can encapsulate hydrophobic drug molecules in the HPE cavities, but also can form hybrid hydrogels with a mechanically tough network and a highly porous interconnected structure upon UV exposure. The physical properties of the resulting hydrogels, such as the microstructure, compressive modulus and drug retainability, as well as cell adhesion and proliferation can be tunable by changing the crosslinking density. The nanocomposite hydrogel showed a spatial control of cell adhesion and controlled release of hydrophobic drug, which is difficult to achieve when using conventional hydrogels made from linear polymers. Wang *et al.* [418] reported a PAMAM dendrimer-based poly(lactic acid)-poly(ethylene glycol)-poly(lactic acid) (PLA-PEG-PLA) hydrogel with a highly porous structure. In comparison with hydrogel without incorporating dendrimer, the nanocomposite hydrogels exhibit reduced swelling, enhanced mechanical stiffness, and better cell adhesion, differentiation, and proliferation. These studies indicated that the hyperbranched dendrimers can be used to reinforce polymeric network and impart hydrogels a stable attachment and a high binding capacity to proteins and drugs. The nanocomposite hydrogels from dendrimers NPs with tailored physicochemical properties exhibited a promising potential to serve as a model for developing advanced materials for various tissue engineering and drug delivery applications.

Overall, the fabrication of composite materials by integrating polymer NPs into hydrogels can not only surmount individual shortcomings, but also generate newborn characters. The hydrogels can preserve the structural integrity and the functionalities of the contained NPs and anchor the NPs at the target site. Meanwhile, NPs entrapped into hydrogel allow a more flexible loading and controlled release of drugs. Apparently, due to the synergistic benefits, mechanically stiff hydrogels with controlled drug delivery characteristics will provide an efficient route to improve the performances and expand application scopes of hydrogels and NPs. However, it was worth pointing out that additional animal experiments are required to examine their behavior *in vivo*, as most of the studies on polymer NPs-based hybrid hydrogels have been performed *in vitro*.

## 4. Conclusions and Future Perspective

During the past several decades, hydrogels and nanoparticles have already exerted a dramatic impact in biological, biomedical, pharmaceutical and diagnostic fields. However, their some intrinsic shortcomings severely restrict their practical applications. Significant efforts have been paid to improve the performance of hydrogels and NPs. The fabrication of composite materials by combining two or more components in a single entity can surmount individual shortcomings and give rise to synergistic functions that are absent in the individual components. The incorporation of NPs in three-dimensional polymeric hydrogel matrix as an innovative means to obtain nanocomposite hydrogels with improved properties and multiple functionalities has gained enormous attention in many areas. On the basis of this review, we can found that various types of NPs, such as carbon-based NPs, silicon-based NPs, metal NPs and polymeric NPs are combined with the polymeric hydrogel network to create multicomponent systems. The porous structure and free space within the hydrogel networks can not only provide an ideal hydrated environment for the stabilization of NPs without aggregations or disintegration and the protection of NPs from degradation or denaturation, but also work as a reservoir to localize NPs at the target site. More importantly, the coatings of hydrogels around the NPs endow a hydration layer, which often is an essential prerequisite for the biomedical and biological applications of inorganic and metal NPs, as the hydration layer can significantly improve the biocompatibility and reduce the cytotoxicity. On the other hand, the incorporation of NPs into hydrogels can not only markedly improve their mechanical, elastic and adhesive properties as well as physicochemical and thermal stability, but also promote the cell attachment and proliferation and improve drug loading capacity and drug release profiles. More significantly, the encapsulation of carbon, metal colloidal particles or quantum dots into polymer hydrogel networks will impart them with exclusive thermal, sonic, optical, electrical or magnetic properties, which are not achieved by individual polymeric systems and are highly appropriate for various applications, especially for therapeutic and diagnostic applications. Apparently, the benefits of the combination of NPs and hydrogels have resulted in generation of a new class of advanced materials with unique properties that have a wide spectral range of biomedical applications, ranging from controlled drug delivery depots, cell and tissue adhesive matrices, wound dressing and tissue engineering scaffolds, stem cell engineering and regenerative medicines, biosensors, actuators and other biomedical devices.

However, despite of the vast potential applications of nanocomposite hydrogels in biomedical fields, there are still lots of challenges to be overcome before they can be applied in clinical use. For example, the improved performance of the nanocomposite hydrogels is mainly ascribed to the enhanced interactions between the NPs and polymer chains. Therefore, apart from particle parameters (size, shape, surface properties of NPs), the quantity and homogeneity of NPs integration are still a matter of concern. To obtain excellent performance, NPs should be abundantly and homogeneously dispersed within the hydrogel matrix. However, due to a large surface area, hydrophobic NPs physically embedded in the hydrophilic polymer matrix often tend to aggregate, leading to the failure of anticipative enhancement of properties. Therefore, there is still a need to elucidate the mechanisms and interactions between NPs and polymer chains inside the nanocomposite hydrogels, and the simple, cost-effective, scalable and reproducible preparations of nanocomposite hydrogels with desirable properties need to be investigated thoroughly. Apparently, the nanocomposite hydrogels combining the individual functions of NPs and hydrogels are ideal candidates for multimodal drug delivery platform with simultaneous capabilities in drug encapsulation, targeting delivery, photothermal therapy and *in vivo* imaging. This synergistic performance is usually not be achieved by an individual. However, the more detailed pharmacological studies need to demonstrate the therapeutic efficacy and biological responses. In addition, most of nanocomposite hydrogels have been performed only *in vitro*. Therefore, in future studies, the detailed performances (gelation time, swelling, elastic modulus, responsivity and functionality), long-term toxicity and biodegradability as well as the biological properties such as protein adsorption, cell adhesion, tissue compatibility and whole-body effect of such hydrogels under *in vivo* conditions should be clearly addressed. In summary, there should be a coordinated and comprehensive research to establish fundamental interactions among nanoparticulate materials, hydrogel matrices and biological systems, before hydrogels will become practical and useful in this exciting field. All these challenges will drive the effective collaboration of scientists from the fields of chemistry, materials, engineering, biology, medicine and nanoscience. We look forward to seeing many exciting research accomplishments in this burgeoning field of bio-related nanocomposite hydrogels.
